# Synergistic effect of fasting-mimicking diet and vitamin C against *KRAS* mutated cancers

**DOI:** 10.1038/s41467-020-16243-3

**Published:** 2020-05-11

**Authors:** Maira Di Tano, Franca Raucci, Claudio Vernieri, Irene Caffa, Roberta Buono, Maura Fanti, Sebastian Brandhorst, Giuseppe Curigliano, Alessio Nencioni, Filippo de Braud, Valter D. Longo

**Affiliations:** 1 University of Milan. Department of Oncology and Hemato-Oncology, Milan, Italy; 2 IFOM, FIRC Institute of Molecular Oncology, Milan, Italy; 3 Medical Oncology Department, Fondazione IRCCS Istituto Nazionale Tumori, Milan, Italy; 4 Department of Internal Medicine, University of Genoa, Genoa, Italy; 5 IRCCS Ospedale Policlinico San Martino, Genoa, Italy; 6 Longevity Institute, Leonard Davis School of Gerontology and Department of Biological Sciences, University of Southern California, Los Angeles, CA USA; 7 Division of Early Drug Development, European Institute of Oncology, IRCCS, Milan, Italy; 8 Present Address: Department of Molecular Biology and Biochemistry, University of California, Irvine, CA USA

**Keywords:** Cancer metabolism, Cancer therapy

## Abstract

Fasting-mimicking diets delay tumor progression and sensitize a wide range of tumors to chemotherapy, but their therapeutic potential in combination with non-cytotoxic compounds is poorly understood. Here we show that vitamin C anticancer activity is limited by the up-regulation of the stress-inducible protein heme-oxygenase-1. The fasting-mimicking diet selectivity reverses vitamin C-induced up-regulation of heme-oxygenase-1 and ferritin in *KRAS*-mutant cancer cells, consequently increasing reactive iron, oxygen species, and cell death; an effect further potentiated by chemotherapy. In support of a potential role of ferritin in colorectal cancer progression, an analysis of The Cancer Genome Atlas Database indicates that *KRAS* mutated colorectal cancer patients with low intratumor ferritin mRNA levels display longer 3- and 5-year overall survival. Collectively, our data indicate that the combination of a fasting-mimicking diet and vitamin C represents a promising low toxicity intervention to be tested in randomized clinical trials against colorectal cancer and possibly other *KRAS* mutated tumors.

## Abstract (web summary)

Fasting diets are emerging as an approach to delay tumor progression and improve cancer therapies. Here, the authors show that the combination of fasting-mimicking diet with vitamin C decreases tumor development and increases chemotherapy efficacy in KRAS-mutant cancer.

## Introduction

Pharmacological doses of vitamin C (ascorbic acid) have been proposed as a potential anticancer therapy according to different pre-clinical and clinical trials^1–3^. Recent studies indicate that *KRAS*-mutant cancers may exhibit higher susceptibility to vitamin C antitumor effects, thus making this generally non-toxic compound a potential weapon against this aggressive tumor type^4,5^. High-dose vitamin C can exert its anticancer effects by pro-oxidant reactions, which cause the formation of hydrogen peroxide and hydroxyl radicals via Fenton chemistry. In turn, these reactive oxygen species (ROS) cause damage to macromolecules, thereby leading to cell death^3,4,6–8^. Vitamin C’s pro-oxidant action is strictly dependent on metal-ion redox chemistry. In particular, free iron was shown to be a key player in vitamin C-induced cytotoxic effects^3,9^.

*KRAS*-mutant cancer cells rely on high levels of ROS and on a labile iron pool (LIP) to sustain their growth^10–12^. Increasing evidence supports the link between ROS production and free iron^10,13,14^. In particular, ROS could release iron from iron-containing proteins, thus expanding the LIP, which in turn could enhance ROS formation through redox chemistry^10,13,14^. Moreover, RAS oncogenic activation further increases cellular iron content by upregulating iron-uptake proteins, such as transferrin receptor (Trf1), and by downregulating iron export and storage proteins, in particular ferritin^10–12^. In this context, the stress inducible protein heme-oxygenase-1 (HO-1), regulates ferritin expression to avoid an excessive free iron content, which can damage macromolecules^15^.

Although high-dose vitamin C could potentially be effective in treating patients with *KRAS* mutated cancers, it is unlikely that this treatment, when used as a monotherapy, would be sufficient to target the molecular heterogeneity and multiple escape mechanisms of these tumors^16^. Therefore, strategies to enhance and expand vitamin C activity in the treatment of *KRAS* mutated cancers are necessary.

We have previously shown that fasting or a fasting-mimicking diet (FMD) reduce tumor progression and sensitize different types of cancer to chemotherapy, while protecting normal cells from chemo-toxic side effects^17,18^. These phenomena are known as “Differential Stress Sensitization” and “Differential Stress Resistance”, respectively^17–21^. The differential effects of fasting on normal (protection) and cancer (sensitization) cells can be  mediated, at least in part, by its effects on the insulin-like growth factor 1 (IGF-1) signaling pathway and on glucose levels^19–22^. However, since fasting remains a challenging option for cancer patients, a more feasible and safer diet whose specific formulation mimics the effects of fasting was developed^23,24^. FMD refers to a plant-based, calorie-restricted, low sugar, low protein, and high-fat dietary composition administered cyclically and alternated with refeeding periods sufficient to prevent or minimize lean body mass loss (the caloric content of the FMD that we used for this study is indicated in the “Methods” session)^24^.

To identify a highly effective but a low toxicity treatment for KRAS-mutant cancers, here we investigate the effect of FMD in potentiating the anticancer activity of vitamin C, alone or in combination with standard chemotherapy with a focus on colorectal cancer (CRC). Our findings reveal that FMD cycles selectively potentiate vitamin C anti-cancer effect against *KRAS*-mutant tumors, both in vitro and in vivo, by reverting the vitamin C mediated upregulation of HO-1. We also provide data showing that the toxic action exerted by FMD and vitamin C is further potentiated by chemotherapy. Together, our data support that cycles of FMD plus pharmacological doses of vitamin C could represent a promising therapeutic opportunity to be tested in the clinic for the treatment of *KRAS* mutated cancers.

## Results

### FMD enhances vitamin C toxicity in *KRAS-*mutant cancer cells

**Fig. 1:**
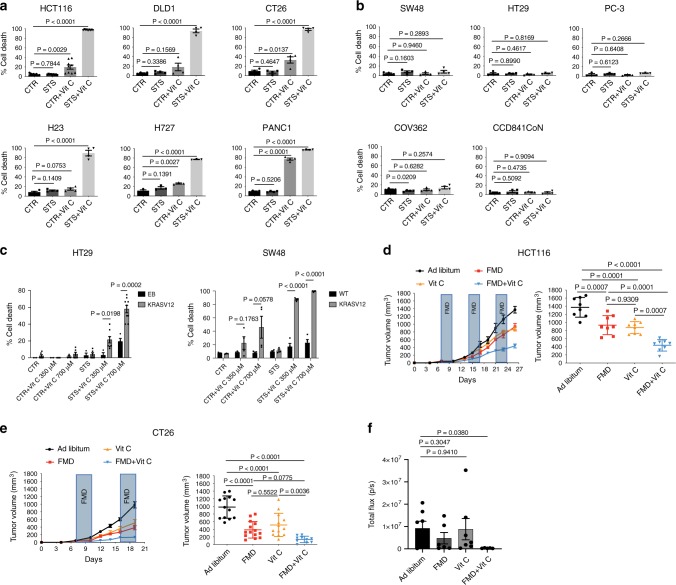
FMD/STS enhances vitamin C anticancer activity in *KRAS*-mutant tumors. **a** Viability of *KRAS*-mutant and **b** *KRAS*-wild-type cancer cells treated for 48 h with STS with or without vitamin C; HCT116 (*n* = 9); DLD1, CT26, H23, and PANC1 (*n* = 4); H727 (*n* = 3); SW48 and HT29 (*n* = 4); PC-3 (*n* = 3); COV362 and CCD841CoN cells (*n* = 4). *P* values were determined by two-sided unpaired *t*-test. DLD1: exact *P* value = 0.0000005; CT26: exact *P* value = 0.00000009; H23: exact *P* value = 0.00001; H727: exact *P* value = 0.000005; PANC1: exact *P* values = 0.0000001 (CTR vs CTR + Vit C), 0.00000000004 (CTR vs STS + Vit C). **c** Viability of HT29 cells infected with empty backbone (EB; *n* = 3, *n* = 4 in STS + Vit C 700 µM) or KRASV12 vector (*n* = 6, *n* = 7 in STS + Vit C 125 µM, *n* = 8 in CTR + Vit C 700 µM and STS + Vit C 350 µM, *n* = 9 in STS + Vit C 700 µM); SW48 WT (*n* = 4) and SW48 KRASV12 (*n* = 4, *n* = 3 in CTR and STS) treated for 48 h with STS with or without vitamin C. *P* values were determined by two-sided unpaired *t*-test. SW48: exact *P* values= 0.000008 (STS + Vit C 350 µM wt vs STS + Vit C 350 µM KRASV12), 0.000005 (STS + Vit C 700 µM wt vs STS + Vit C 700 µM KRASV12). **d** Tumor growth of HCT116-derived xenograft (*n* = 8) and **e** CT26-derived allografts (*n* = 13 in Ad libitum and Vit C, *n* = 14 in FMD and *n* = 10 in FMD + Vit C). Tumor volumes at multiple time points (left) and before euthanasia (right) are presented. *P* values were determined by One-way ANOVA with Tukey’s post analysis. HCT116: exact *P* value = 0.000000002 (Ad libitum vs FMD + Vit C); CT26: exact *P* values = 0.0000000001 (Ad libitum vs FMD + Vit C), 0.00008 (Ad libitum vs Vit C), 0.0000007 (Ad libitum vs FMD). **f** Tumor growth of CT26-luc-derived orthotopic model (*n* = 7 in Ad libitum and Vit C, *n* = 6 in FMD and *n* = 5 in FMD + Vit C). Total photon effluxes over tumor regions were measured. *P* values were determined by two-sided unpaired *t*-test. All data are represented as mean ± SEM, *n* = independent experiments.

We investigated whether the fasting/FMD potentiates the anti-cancer effect of vitamin C against different *KRAS*-mutant cancer models. Human (HCT116, DLD-1) and murine (CT26) *KRAS*-mutant CRC cell lines, as well as *KRAS*-mutant lung cancer (H23, H727) and pancreatic ductal adenocarcinoma (PANC1) cells, were grown in control medium (1 g/L glucose and 10% serum; CTR) or in a FMD-like medium (0.5 g/L glucose and 1% serum), here referred to as short-term starvation condition (STS), which mimics the reduction of extracellular glucose and growth factor concentrations that occurs during prolonged (>48 h) fasting or FMD in vivo, with or without pharmacological concentrations of vitamin C (≥0.3 mM). Consistent with recent findings^4^, *KRAS*-mutant tumor cells were more susceptible to vitamin C compared with *KRAS*-wild-type cancer cells (Fig. 1a, b). When cancer cells were grown under STS conditions before and during treatment, vitamin C-mediated toxicity was strongly enhanced (Fig. 1a). Conversely, *KRAS*-wild-type CRC (SW48, HT29), prostate cancer (PC-3), ovarian cancer (COV362) cell lines and a normal colon cell line (CCD841CoN) were resistant to vitamin C when used both as a single agent and in combination with STS (Fig. 1b). In agreement with the selective toxicity of STS + vitamin C in *KRAS*-mutant tumor cells, we found that HT29 and SW48 cells genetically modified to express the active form of KRAS were more susceptible to STS + vitamin C compared with their wild-type isogenic counterpart (Fig. 1c and Supplementary Fig. 1).

Consistent with our in vitro results, we found that FMD cycles combined with daily vitamin C treatment (4 g per kg twice a day) were effective in delaying the progression of *KRAS* mutated tumors in different mouse models (Fig. 1d–f). In particular, weekly cycles of a three days FMD were sufficient to reduce *KRAS* mutated tumor growth to the same extent as high-dose vitamin C (Fig. 1d, e). Notably, weekly FMD and daily vitamin C showed the best therapeutic outcome in reducing CRC progression in xenograft and syngeneic mouse models as well as in an orthotopic model (Fig. 1d–f and Supplementary Fig. 2a).

Furthermore, the FMD-vitamin C combination was safe and well tolerated in both mouse strains, as indicated by mouse body weight loss, which did not exceed 20% and was rapidly recovered upon refeeding (Supplementary Fig. 2b).

### ROS mediate sensitization to vitamin C

We previously showed that fasting/FMD sensitizes different types of cancer cells to chemotherapy through a mechanism that involves increased ROS production^17,25^. ROS, including H_2_O_2_ and superoxide, generated as by-products of normal metabolism, cause damage to DNA, lipids and proteins^26^.

**Fig. 2:**
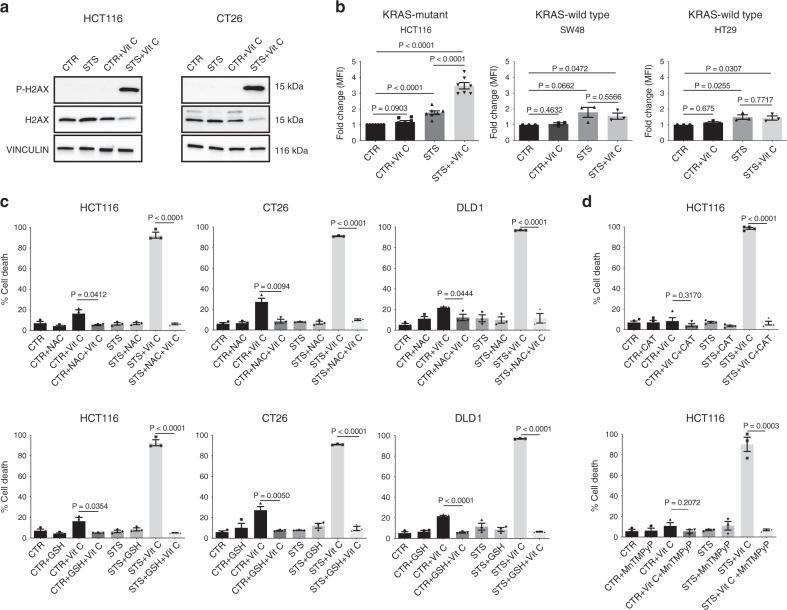
ROS mediate *KRAS*-mutant cancer cell sensitization to vitamin C via STS. **a** Detection of phosphorylated H2AX levels in HCT116 and CT26 by western blotting, total H2AX and VINCULIN as loading control (*n* = 3). **b** CellROX-Deep-Red ROS detection by flow cytometry in HCT116 (*n* = 7), SW48 and HT29 (*n* = 3). MFI: mean fluorescence intensity. *P* values were determined by two-sided unpaired *t*-test. HCT116: exact *p* value = 0.00000004 (CTR vs STS + Vit C), 0.00003 (CTR vs STS), 0.00001 (STS vs STS + Vit C). **c** Viability of HCT116 (*n* = 3), CT26 (*n* = 3), DLD1 (*n* = 3) cells treated for 48 h with STS with or without vitamin C, glutathione (GSH), N-acetyl cysteine (NAC) and **d** catalase (CAT, *n* = 4) or the superoxide dismutase (SOD)/catalase mimetic MnTMPyP (HCT116, *n* = 3). *P* values were determined by two-sided unpaired *t*-test. HCT116 in (**c**): exact P values= 0.000009 (STS + Vit C vs STS + NAC + Vit C), 0.000007 (STS + Vit C vs STS + GSH + Vit C); CT26: exact *P* values = 0.00000007 (STS + Vit C vs STS + NAC + Vit C), 0.000002 (STS + Vit C vs STS + GSH + Vit C); DLD1: exact *P* values = 0.00005 (STS + Vit C vs STS + NAC + Vit C), 0.000000003 (STS + Vit C vs STS + GSH + Vit C), 0.00008 (CTR + Vit C vs CTR + GSH + Vit C). HCT116 in (**d**): exact *P* value = 0.000000007 (STS + Vit C vs STS + Vit C + CAT). All data are represented as mean ± SEM, *n* = independent experiments.

Recent studies have shown that *KRAS* mutations promote metabolic reprogramming to sustain high-proliferation rates, accompanied by a higher oxidative state compared with *KRAS*-wild-type cells^11,12,27,28^. Thus, we hypothesized that the higher oxidative state of *KRAS*-mutant tumors may underlie the selective mechanism of FMD + vitamin C toxicity. Notably, the combination of STS and vitamin C strongly induced DNA damage in CT26 and HCT116 cells, as indicated by the phosphorylation of the histone H2AX (Fig. 2a), suggesting that oxidative stress may participate in mediating this cytotoxic effect. Indeed, the combination of STS and vitamin C selectively exacerbated ROS production in *KRAS*-mutant tumor cells (Fig. 2b and Supplementary Fig. 3a). Consistent with the vitamin C-dependent production of H_2_O_2,_ we observed the same selective increase in ROS in *KRAS*-mutant cancer cells grown in STS condition and treated with H_2_O_2_ (Supplementary Fig. 3b).

To directly assess whether increased ROS production is a causative or a secondary event in STS-induced sensitization to vitamin C, we evaluated the effect of different antioxidants on STS + vitamin C toxicity (Fig. 2c, d and Supplementary Fig. 3c). Glutathione (GSH) and N-acetyl cysteine (NAC), as well as prior exposure to membrane-impermeable catalase (CAT) or membrane-permeable superoxide dismutase (SOD)/catalase mimetic MnTMPyP were able to revert the STS + vitamin C induced cell death (Fig. 2c, d).

Collectively, these findings indicate that ROS production and redox alterations represents a central mechanism through which the STS and vitamin C combination selectively kills *KRAS* mutated cancer cells.

### Iron is involved in FMD-mediated toxicity

**Fig. 3:**
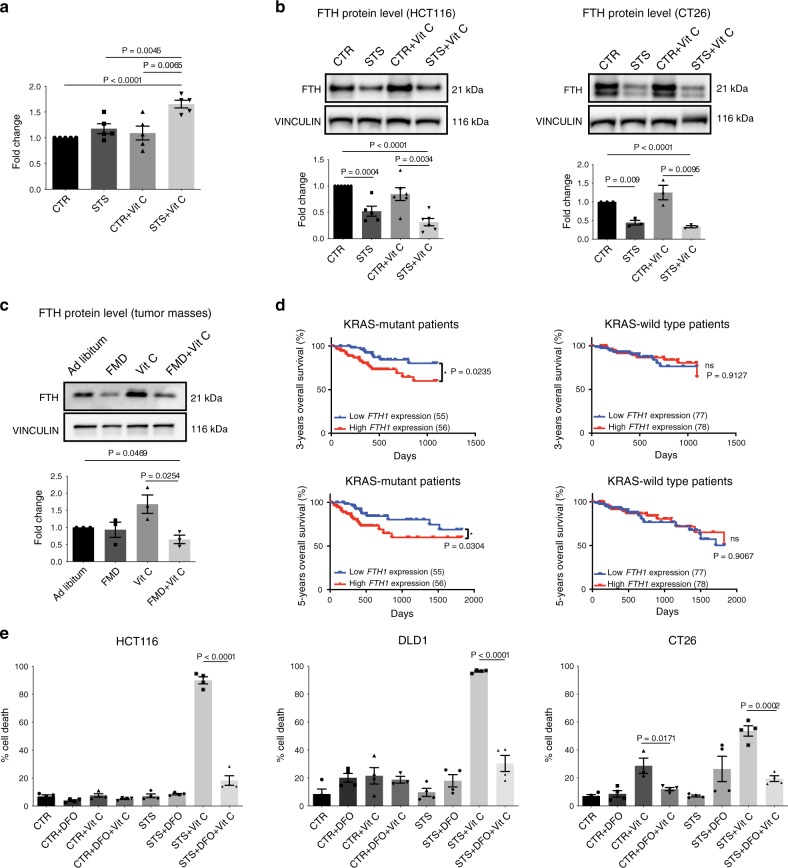
Iron is involved in FMD + vitamin C toxicity selectively in *KRAS* mutated cancer cells. **a** Intracellular free iron (Fe^2+^) measurement, relative to CTR cells, of HCT116 treated with STS with or without vitamin C (*n* = 5). *P* values were determined by two-sided unpaired *t*-test. Exact P value= 0.00002 (CTR vs STS + Vit C). **b** Detection of ferritin (FTH) protein expression by western blot in HCT116 (*n* = 6, *n* = 5 in STS), CT26 cells (*n* = 3) and **c** HCT116-derived tumor masses (*n* = 3). VINCULIN as loading control. *P* values were determined by two-sided unpaired *t*-test. HCT116 exact *P* value = 0.000002 (CTR vs STS + Vit C); CT26 exact *P* value = 0.00002 (CTR vs STS + Vit C). **d** Three-year (left panel) and 5-year (right panel) overall survival of patient bearing *KRAS*-mutant (left) and *KRAS*-wild-type (right) tumors collected from The Cancer Genome Atlas Database (TCGA) and stratified according to intratumor *FTH1* mRNA expression levels. P values were determined by Wilcoxon matched-pairs signed rank test. **e** Viability of HCT116, DLD1 and CT26 cells in response to STS, vitamin C, or their combination with or without desferrioxamine (DFO) (*n* = 4, *n* = 3 in DLD1- CTR + DFO + Vit C and CT26- CTR + Vit C). *P* values were determined by two-sided unpaired *t*-test. HCT116: exact *P* value = 0.000003 (STS + Vit C vs STS + DFO + Vit C); DLD1: exact *P* value = 0.00003 (STS + Vit C vs STS + DFO + Vit C). All data are represented as mean ± SEM, *n* = independent experiments.

A large body of evidence shows that the mechanism underlying vitamin C’s anti-cancer effects relies on H_2_O_2_ production and that the LIP plays a fundamental role in this process^3,6,7^. In the presence of free iron, high H_2_O_2_ levels have pro-oxidant effects in part through the generation of hydroxyl radicals via Fenton reaction and the induction of oxidative damage^3,7^. Since the combination of FMD/STS and vitamin C increased ROS levels in *KRAS*-mutant cancer cells, we investigated whether this correlates with an increased pool of labile ferrous iron. In HCT116 cells, ferrous ion (Fe^2+^) levels were significantly increased upon STS and vitamin C co-treatment compared with all other conditions (Fig. 3a), suggesting a potential role of iron in mediating this effect. Ferritin, the main protein involved in iron binding and storage, regulates the intracellular LIP and its downregulation has been shown to increase LIP in *KRAS*-mutant cancer cells^10–12^. Thus, we measured the levels of the heavy subunit of ferritin (FTH), which is responsible for the iron storage through its ferroxidase activity^10^. Consistent with the increase in ferrous iron, we found that STS, alone or in combination with vitamin C, downregulated FTH protein expression selectively in *KRAS*-mutant cancer cells. On the other hand, vitamin C reversed STS-induced ferritin downregulation in *KRAS*-wild-type tumor cells (Fig. 3b, Supplementary Fig. 4). These in vitro results were also confirmed in vivo, where FMD cycles combined with vitamin C treatment downregulated FTH protein expression in HCT116-derived tumors (Fig. 3c).

Consistent with a sensitizing effect of FTH downregulation in *KRAS* mutant cancer cells, our analysis of CRC patient-survival data acquired from The Cancer Genome Atlas Database (*TCGA*), showed that patients with *KRAS* mutated tumors and low intratumoral ferritin transcriptional level had a longer 3- and 5-year overall survival when compared with patients whose tumors expressed high ferritin level (Fig. 3d). This association, which was not observed for wild type KRAS tumors, supports the role for FMD + vitamin C as a strategy to maintain low ferritin levels and increase ROS to treat *KRAS* mutated tumors. Notably, these data represent less than 200 patients; therefore, analyses of larger patient populations are required to better understand the role of ferritin expression, or activity, in *KRAS* mutated tumors progression.

To assess whether the alteration in cellular iron content contributes to STS-mediated sensitization to vitamin C, *KRAS*-mutant CRC cells grown in STS conditions were treated with the iron chelator desferrioxamine (DFO) before vitamin C exposure. Consistent with our hypothesis, DFO treatment preceding vitamin C exposure rescued vitamin C-induced cell cytotoxicity (Fig. 3e), thus confirming that the increase in intracellular free iron mediated by FMD/STS and vitamin C is, at least in part, responsible for their cytotoxic effect.

### FMD reverses the effect of vitamin C on HO-1

Several studies suggested a potential role of ferritin in protecting cells from oxidative damage through the sequestration of intracellular free iron^10–12^. Among enzymes promoting ferritin expression, the stress-inducible HO-1 has been implicated in promoting cell survival during cell exposure to oxidative insults^29,30^. Since the FMD/STS downregulates the FTH protein expression level, we investigated whether HO-1 is implicated in FTH regulation in response to STS and vitamin C treatment. In one recent study from our group, the FMD sensitized breast cancer cells to chemotherapy in part by downregulating HO-1, further supporting a possible role of this stress-inducible protein in mediating FMD beneficial effects^31^.

**Fig. 4:**
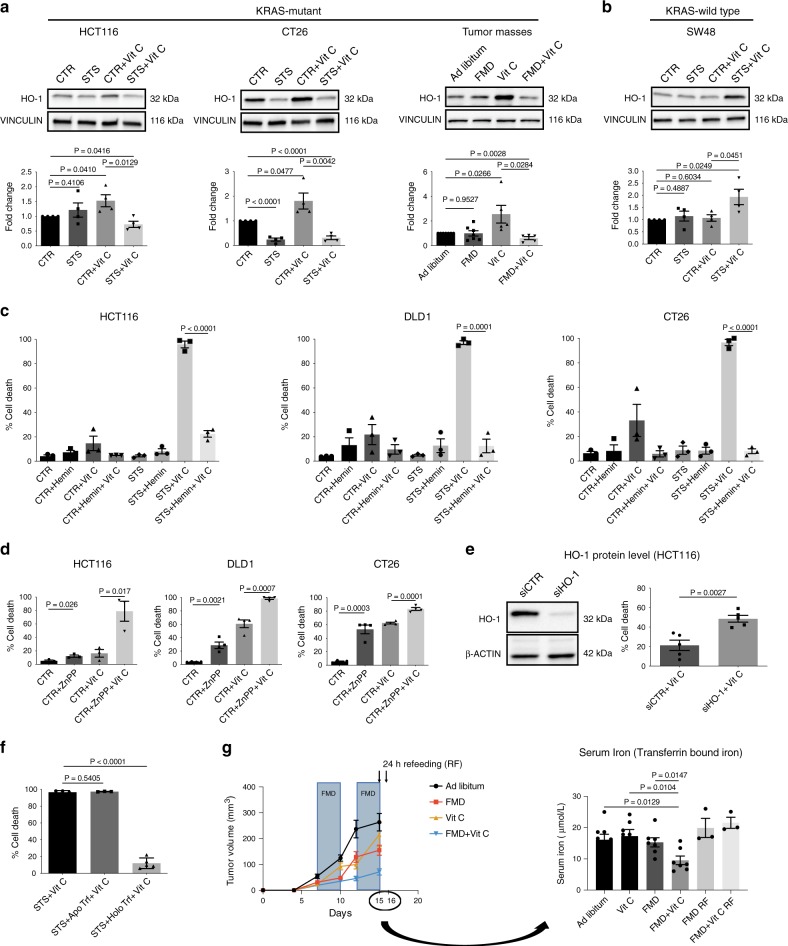
HO-1 modulation and iron-bound transferrin are the key players in FMD-dependent sensitization to Vitamin C. **a**, **b** Western blotting detection of HO-1 expression level in *KRAS*-mutant HCT116 (*n* = 4), CT26 (*n* = 4) and HCT116-derived tumor masses (*n* = 5 in Vit C and FMD + Vit C, *n* = 7 in Ad libitum and FMD) and *KRAS*-wild-type SW48 cancer cells (*n* = 4). VINCULIN as loading control. Representative blots and quantifications are shown. *P* values were determined by two-sided unpaired *t*-test. CT26: exact *P* values = 0.00002 (CTR vs STS), 0.00009 (CTR vs STS + Vit C). **c** Viability of DLD1, HCT116, and CT26 cells treated with STS with or without vitamin C, hemin (*n* = 3) or **d** zinc protoporphyrin (ZnPP; HCT116, *n* = 3; DLD1 and CT26, *n* = 4). *P* values were determined by two-sided unpaired *t*-test. HCT116 in (**c**): exact *P* value = 0.00005 (STS + Vit C vs STS + Hemin + Vit C), CT26 in (**c**): exact *P* value= 0.00001 (STS + Vit C vs STS + Hemin + Vit C). **e** Western blot (left) and viability (right) of HCT116 cells transfected with control siRNAs (siCTR) or anti-HO-1 siRNAs (siHO-1), *n* = 5. *P* values were determined by two-sided unpaired *t*-test. **f** Viability of HCT116 cells grown in STS medium with apo-transferrin (ApoTrf) or holo-transferrin (HoloTrf) with or without vitamin C (*n* = 4 in STS, *n* = 3 in ApoTrf and *n* = 5 in HoloTrf). *P* values were determined by two-sided unpaired *t*-test. Exact *P* value = 0.00000003 (STS + Vit C vs STS + HoloTrf + Vit C). **g** Quantification of transferrin bound iron in mouse blood serum (right) of HCT116-engrafted NSG mice fed ad libitum or subjected to FMD cycles, and treated with or without vitamin C (tumor growth, left). Black arrows indicate blood serum collection: upon the last FMD cycle and 24 h after refeeding (RF). *P* values were determined by two-sided unpaired *t*-test (*n* = 8 mice in Ad libitum, *n* = 7 in FMD, FMD + Vit C, Vit C, *n* = 3 in FMD RF, FMD + Vit C RF). All data are represented as mean ± SEM, *n* = independent experiments.

To test our hypothesis, we evaluated the association between HO-1 expression and FTH induction. To this end, we treated HCT116 cells with the HO-1-activator hemin and verified that hemin increases both HO-1 and FTH protein levels under CTR and STS growing conditions (Supplementary Fig. 5). Furthermore, we found that treatment with vitamin C significantly upregulated HO-1, while FMD/STS reversed this effect both in vitro and in vivo in *KRAS*-mutant cancer cells (Fig. 4a and Supplementary Fig. 6a). In *KRAS*-wild-type cancer cells HO-1 levels were not altered upon vitamin C administration under CTR condition, and the combination of vitamin C and STS did not downregulate but instead induced HO-1 protein expression level (Fig. 4b, Supplementary Fig. 6b).

Collectively, these findings indicate that the differential regulation of HO-1 and the resulting effect on ferritin/iron pathway mediate the FMD-dependent sensitization to vitamin C selectively in a *KRAS*-mutant background.

The ability of the FMD to prevent vitamin C-induced HO-1 upregulation in *KRAS*-mutant cancer cells prompted us to investigate whether HO-1 levels affect the sensitivity of tumor cells to the combination treatment. Consistent with this hypothesis, the HO-1 activator hemin protected human and murine *KRAS*-mutant CRC cells from vitamin C-induced cell death, alone or in combination with STS (Fig. 4c). On the other hand, the HO-1 inhibitor zinc protoporphyrin (ZnPP) made *KRAS*-mutant CRC cells more susceptible to vitamin C in nutrient-rich condition (CTR) (Fig. 4d). Consistent with these findings, HO-1 knockdown in HCT116 cells also increased cancer cell death upon vitamin C exposure in CTR condition (Fig. 4e). Thus, overall these results support the role of HO-1 in regulating *KRAS*-mutant cancer cell sensitivity to vitamin C.

To characterize how FMD/STS affects HO-1 levels and modulate cancer cell sensitivity to vitamin C, we analysed the effect of glucose or serum deprivation. Interestingly we found that glucose, serum growth factors and amino acids were not responsible for the STS-dependent enhancement of vitamin C toxicity (Supplementary Fig. 7a). Addition of holo-transferrin (iron-bound form) but not apo-transferrin (iron-free form) reversed STS + vitamin C-mediated toxicity and HO-1/FTH axis downregulation (Fig. 4f; Supplementary Fig. 7b). These results are consistent with the concept that iron levels in the serum are important in mediating the STS effect. In fact, in agreement with our in vitro findings, in vivo FMD + vitamin C reduced blood levels of transferrin bound iron (Fig. 4g). Our data further support the role of iron as a key factor in the serum whose reduction was responsible for the synergism of FMD and vitamin C.

### FMD and vitamin C potentiate oxaliplatin cytotoxic effects

A number of studies described the tolerability and potential efficacy of high-dose vitamin C as an adjuvant treatment during chemotherapy^2,3,32,33^. In addition, our group has recently shown the effectiveness of fasting or FMD cycles in combination with chemotherapy to reduce tumor growth in a wide range of cancer types compared with standard chemotherapy alone^17,31^.

**Fig. 5:**
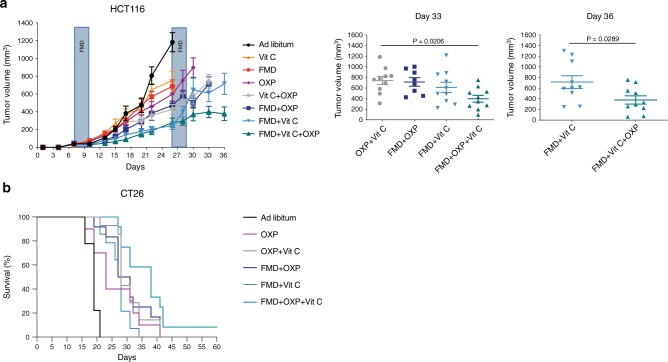
FMD, vitamin C and OXP triple treatment delays tumor progression and extends survival. NSG and BALB/c mice were subcutaneously injected with HCT116 cells and CT26 cells, respectively. Mice were fed ad libitum or subjected to FMD cycles, and treated with or without vitamin C or oxaliplatin (10 mg/kg). **a** HCT116 tumor progression (left) and volume at day 33 and 36 (right), respectively (*n* = 10 in Ad libitum, FMD, FMD + Vit C, Vit C + OXP, FMD + Vit C + OXP, *n* = 8 in FMD + OXP, *n* = 9 in OXP, *n* = 11 in Vit C). *P* values were determined by One-way ANOVA with Tukey’s post analysis (day 33) and two-sided unpaired *t*-test (day 36). Data are represented as mean ± SEM. **b** BALB/c with CT26 survival curves (*n* = 9 in Ad libitum, *n* = 10 in OXP, *n* = 12 in FMD + OXP and FMD + OXP + Vit C, *n* = 14 in OXP + Vit C and FMD + Vit C). *P* values were determined by Log-rank (Mantel-Cox) test (Ad libitum vs OXP, p = 0.0040; OXP vs FMD + Vit C, *p* = 0.7177; OXP vs FMD + OXP + VitC, *p* = 0.0114; FMD + OXP vs FMD + OXP + Vit C, *p* = 0.0488; OXP + Vit C vs FMD + OXP + Vit C, *p* = 0.0345; FMD + OXP + Vit C vs FMD + Vit C, *p* = 0.0003).

Drawing from our previous data, we investigated whether FMD + vitamin C would sensitize *KRAS*-mutated cancer cells to the pro-oxidant action of chemotherapy in vivo, possibly by increasing cellular oxidative stress. We chose oxaliplatin because it is one of the most effective cytotoxic compounds used in the adjuvant and advanced setting of CRC treatment^34^. Of note, the FMD + vitamin C combination was as effective as oxaliplatin + FMD or oxaliplatin + vitamin C, supporting the powerful action of these non-toxic combinations in halting tumor growth (Fig. 5a).

Moreover, triple treatment (FMD + vitamin C + chemotherapy) was the most active therapeutic intervention in delaying tumor progression in a mouse xenograft and in extending survival in a syngeneic model (Fig. 5a, b and Supplementary Fig. 8). These results indicate that chemotherapy can further potentiate the effects of FMD + vitamin C against *KRAS* mutated cancers.

## Discussion

In preclinical experiments, fasting reduces tumor progression and sensitizes different tumor types to chemotherapy, while protecting normal cells through a mechanism that can involve the reduction of blood IGF-1 and glucose levels^17,19,22^. Oxidative stress also contributes to the anticancer properties of fasting^17,25^.

Since prolonged water-only fasting remains a challenging option for cancer patients, FMDs have been recently proposed as more feasible and equally effective interventions^23,24,31^.

Here, we focused on the identification of a low-toxicity treatment consisting of the combination of FMD cycles with pharmacological dosages of vitamin C for the treatment of *KRAS* mutated cancers. Recently, Yun and colleagues reported that high-dose vitamin C is selectively toxic to *KRAS* and *BRAF* mutated CRC cells, proposing that vitamin C could have therapeutic applications in the treatment of these aggressive neoplasms^4^. This effect is due to an enhancement in the uptake of the oxidized form of vitamin C (DHA) via GLUT1 glucose transporter, which is upregulated in these highly glycolytic cancer cells. Here, we found that the anticancer effect of vitamin C can be potentiated in different in vitro and in vivo mouse models by STS/FMD treatment selectively in *KRAS*-mutant cancers, through a mechanism that is independent of glucose, but that involves differential regulation of HO-1. Of note, HO-1 is frequently overexpressed in several tumors in response to chemotherapy, thus representing a potential resistance mechanism and poor prognostic factor in some cancer types^35–38^. Our data suggest that unlike for *KRAS*-mutated cancer cells, where FMD reverts the vitamin C-mediated HO-1 upregulation, *KRAS*-wild-type tumors are more resistant to oxidative insults, possibly because of their ability to rapidly neutralise radical species^4,11,12,27^. The differential modulation of HO-1 expression in *KRAS*-mutant and *KRAS*-wild-type cells can, at least in part, explain the differential response to oxidative damage and thus the sensitivity to vitamin C (Supplementary Fig. 9). The unresponsiveness of *KRAS*-wild-type cells to vitamin C + fasting/FMD does not mean that either vitamin C or fasting/FMD cannot contribute to toxicity against this type of cancer cells, since combination with additional treatments such as chemotherapy could yield additive effects as shown for many other cancer models^17^.

An analysis of TCGA database indicated that patients with *KRAS* mutated CRC but not wild-type *KRAS* CRC expressing low levels of ferritin displayed longer 3- and 5-year survival when compared with patients bearing tumors with high ferritin levels, supporting a role of intracellular iron and free radical scavenging mechanisms in protecting CRC cells also in patients. The current results pointing to the iron binding and transporting proteins as mediators of cancer cell resistance to therapy, together with our previous works, support that FMD cycles, by altering a wide range of nutrients, growth factors, plasma and cellular proteins and metals, are able to affect a wide range of escape routes required for the survival or resistance acquisition of different tumors. The high toxicity of the combination of the FMD together with vitamin C against *KRAS* mutated tumors, provides a clear example of how a non-toxic therapy combining both wide-acting (FMD) and targeted (vitamin C) antitumor mechanisms can be as or more effective in delaying cancer progression in mice as/than standard antitumor therapies that are much more toxic to normal cells and organs.

These data, together with additional published and ongoing clinical studies, indicate that FMD cycles in combination with a variety of therapies could represent a promising non-toxic strategy for the treatment of different cancer types, paving the way for conducting clinical trials to test FMD plus vitamin C in combination with standard of care for the treatment of *KRAS*-mutant cancers.

## Methods

### Cell lines and culture conditions

HCT116, HT29, NCI-H23, and PC-3 cells were obtained from NCI 60 panel; CT26 and CCD84CoN cells were purchased from ATCC; DLD1 cell line was purchased from DSMZ; SW48, NCI-H727, PANC-1, and COV362 cells were purchased from ECACC. SW48 *KRAS* G12D/ + cell lines were purchased from Horizon. CT26-luc cells for in vivo experiment were purchased from GenTarget Inc (SC061-LG). All cell lines were maintained in Dulbecco’s Modified Eagle Medium (DMEM) (Life Technologies, Cat. #: 10566) supplemented with 10% FBS (Biowest, Cat. #: S1810), 1% non-essential amino acids (Biowest, Cat. #: X-0557), and 1% penicillin/streptomycin (Biowest, Cat. #: L0022). All cells were tested for mycoplasma contamination routinely. Cells were maintained in a humidified, 5% CO_2_ atmosphere at 37 °C. For FMD-like condition experiments, cells were grown in DMEM medium without glucose (DMEM no glucose, Life Technologies, Cat. #: 11966025) supplemented with 0.5 g/L glucose (Sigma-Aldrich, Cat. #: G8769) and 1% FBS, referred as Short-Term starvation medium (STS). For mimicking standard condition, cells were grown in DMEM medium without glucose (DMEM no glucose, Life Technologies, Cat. #: 11966025) supplemented with 1 g/L glucose (Sigma-Aldrich, Cat. #: G8769) and 10% FBS, referred as control medium (CTR).

### Reagent preparations

Oxaliplatin was kindly provided by the IEO hospital pharmacy (Milan), stock solution (5 mg/mL) was dissolved in saline solution for injections. Sodium ascorbate was purchased from Sigma-Aldrich (Cat. #: A4034) and was dissolved in sterile saline, stock solutions of 20 mg/mL or 240 mg/mL were prepared for in vitro and in vivo experiments, respectively. Vitamin C was freshly prepared each time before use. Reduced glutathione (GSH) was purchased from Sigma-Aldrich (Cat. #: G6013) and dissolved in sterile water to a final concentration of 32.5 mM (stock solution), stock solutions were stored at -20 °C. N-acetyl cysteine (NAC) was purchased from Sigma-Aldrich (Cat. #: A9165) and dissolved in sterile deionized water to a final concentration of 100 mM (stock solution), stock solutions were freshly prepared for each experiment. DFO was purchased from Sigma-Aldrich (Cat. #: D9533) and dissolved in sterile deionized water to final concentration of 40 mg/mL, stock solutions were stored at −20 °C. Hemin was purchased from Sigma-Aldrich (Cat. #: 51280) and dissolved in 1.4 M NH_4_OH (Sigma-Aldrich, Cat. #: 221228) to a final concentration of 25 mg/mL (stock solution), stock solutions were stored at +4 °C. Zinc protoporphyrin (ZnPP) was purchased from Sigma-Aldrich (Cat. #: 282820) and dissolved in DMSO to a final concentration of 25 mg/mL, stock solutions were stored at −20 °C. Catalase (Cat) from bovine liver (2000–5000 U/mL) was purchased from Sigma–Aldrich (Cat. #: C1345) and dissolved in 50 mM potassium phosphate buffer to a final concentration of 5000 U/mL, stock solutions were freshly prepared for each experiment. MnTMPyP (superoxide dismutase mimetic) was purchased from Merck Millipore (Cat. #: 475872) and dissolved in sterile deionized water to a final concentration of 697 µM, stock solutions were stored at −20 °C.

Hydrogen peroxide was purchased from Sigma-Aldrich (Cat. #: H1009), stock solution was prepared to a final concentration of 200 mM.

### Viability assay

For cell death measurement, cells were seeded in 12-well plates at a concentration in a range of 20,000 to 150,000 cells according to the cell line, so that at the moment of vitamin C treatment, cells reach 40% of confluence. 24 h after seeding, cells were rinsed twice in PBS and then grown in CTR or STS medium. After 24 h, media were refreshed to ensure that glucose and serum levels were not completely exhausted, and after medium pH stabilization at 37 °C and 5% CO_2_ atmosphere, cells were treated with 350 μM vitamin C or vehicle for the next 24 h. For experiments with anti-oxidant agents, cells were treated with 5 mM glutathione, and 5 mM N-acetyl cysteine, together with vitamin C. For experiments with DFO, 500 μM DFO was added in CTR and STS media 6 h before vitamin C treatment for HCT116 cells or 12 h before DLD1 and CT26. After the specific incubation time, cells were washed twice in PBS to evaluate only the intracellular effect of DFO in chelating iron and to avoid possible interactions with chemical components in the growth medium, and then, CTR or STS fresh medium and vitamin C were added for the next 24 h for HCT116 and DLD1 cells, and 9 h for CT26. For hydrogen peroxide scavenging experiments, cells were treated with catalase (CAT) from bovine liver (50 U/mL) 1 min before vitamin C was provided as previously described^3^ or with MnTMPyP (50 µM), 2 h before vitamin C administration.

For HO-1 activation experiments, cells were treated with hemin at a concentration of 20 μM, 3 h before vitamin C was provided. For HO-1 inhibition experiments, cells were treated with zinc protoporphyrin (ZnPP) at a concentration of 20 μM, 3 h before vitamin C was provided. For medium dissection experiments, cells were grown in the following media for a total of 48 h:

-Low concentration of glucose (0.5 g/L) and standard concentration of serum (10% FBS);
-low concentration of serum (1% FBS) and standard concentration of glucose (1 g/L);
-low concentration of glucose (0.5 g/L) and 10% dialyzed FBS;
-STS medium supplemented with IGF-1 (PeproTech, Cat. #: 100-11, 250 ng/ml) or EGF (Biomol, Cat. #: BPS-90201-3, 200 ng/mL) or insulin (Sigma-Aldrich, Cat. #: 11376497001, 200 ng/mL) or their combination with or without glucose (1 g/L);
-STS supplemented with essential amino acids, supplemented as 2× concentration compared with standard medium (Life Technologies, Cat. #: 11130) or non-essential amino acids (Life Technologies, Cat. #: 11140050, 1 mM);
-STS medium supplemented with apo-transferrin (Sigma Aldrich, Cat. #: T2252, 0.3 mg/ml) or holo-transferrin (Sigma Aldrich, Cat. #: T0665, 0.3 mg/mL).

After 24 h media were refreshed and, after medium pH stabilization at 37 °C and 5% CO_2_, cells were treated with vitamin C (350 µM). At the end of the experiment cells were harvested by trypsinization, centrifuged and resuspended for a final concentration of 1 × 10^6^ cells per mL. Cell viability was measured by Muse viability assay kit or Erythrosine B exclusion assay.

For Muse viability assay, cell suspension and Muse viability reagent (Merck Millipore, Cat. #: MCH100102) are mixed in 1:10 ratio and, after 5 min of incubation in the dark, viability was analysed by Muse cell analyser. Data are expressed as percentage of dead cells.

For erythrosine B exclusion assay, cell suspension was diluted 1:1 with erythrosin B 0.1% in PBS (Sigma-Aldrich, Cat. #: 200964), then cells were counted in a Bürker chamber, and percentage of cell death was calculated as the number of Erythrosin B-positive cells with respect to the total number of cells.

### CellTiter96 AQ_eous_ proliferation assay

HT29 cells infected with empty backbone (EB) or KRAS G12V plasmid were seeded in 96 well plate in CTR medium. After 24 h, cells were rinsed twice in PBS and CTR or STS medium was added. After 24 h, media were refreshed to ensure that glucose and serum levels were not completely exhausted and, after medium pH stabilization at 37 °C and 5% CO_2_ atmosphere, cells were treated with 350 μM vitamin C or vehicle for the next 24 h.

Viability was measured by CellTiter96 AQ_UEOUS1_ (Promega) according to the manufacturer’s instructions.

### ROS measurement

For ROS measurement, cells were seeded in 100 mm Petri dish (in a range of 4 × 10^5^ to 3 × 10^6^ cells according to the cell line in CTR medium). After 24 h cells were rinsed twice in PBS and CTR or STS medium was added. 24 h later, cells were trypsinized, resuspended in their respective media, and treated with 1 mM sodium ascorbate or hydrogen peroxide (200 µM) and 1 µM CellROX deep red reagent in the dark, for 30 min at 37 °C and 5% CO_2_. CellROX probe exhibits a fluorescence excitation at 640 nm and fluorescent emission at 665 nm (deep red). Then, fluorescence was immediately analyzed by flow cytometry (Attune NxT flow cytometer). Data were processed by Kaluza analysis software (Beckman coulter, version 2.0). Data were expressed as fold change of the median fluorescent intensity (MFI) of each treated sample versus the MFI of control sample. A detailed description of gating strategy is provided in Supplementary Fig. 10.

### Intracellular ferrous iron detection

Intracellular ferrous ions (Fe^2+^) were measured using Iron Assay Kit (Cat. #: ab83366). Cells were seeded in 100 mm petri dish (4 × 10^5^ cells) in CTR medium. After 24 h cells were rinsed twice in PBS and CTR or STS medium was added. 24 h later, medium was refreshed and cells were treated with 350 µM vitamin C or vehicle for 3 h. Next, cells were lysed in iron assay buffer and intracellular ferrous iron level was measured according to the manufacturer’s protocol. Absorbance was recorded at the wavelength of 593 nm using a microplate reader (Infinite M200 TECAN). Data are expressed as fold change versus control sample.

### RNA interference with siRNA oligonucleotides

RNA interference was carried out on HCT116 using Lipofectamine RNAiMAX (Invitrogen, Cat. #: 13778150) following the supplier’s protocol. HCT116 cells were seeded the day before transfection and transfected at 50–60% confluence with the indicated siRNA oligonucleotides (25 nM). The following oligonucleotides were used: ON-TARGETplus human *HO-1* siRNA (pool of four siRNA) and ON-TARGETplus non-targeting pool as a negative control (Dharmacon). Knockdown efficiency was assessed by western blot analysis.

### Retroviral transduction

pBABE-puro (empty backbone; EB) and pBABE-Puro-KRas V12 were purchased from Addgene (#1764 and #9052). For retroviral transduction, 1 × 10^6^ Phoenix cells were plated in 60 mm Petri dishes and allowed to adhere for 24 h. Thereafter, cells were transfected with 4 μg of plasmid DNA using TransIT-293 (Mirus Bio) according to the manufacturer’s instructions. Viral supernatants were harvested after 36, 48, 60, and 72 h and used to infect HT29 cells (4 × 10^5^) in 100 mm Petri dishes in the presence of 5 μg/mL protamine sulfate. Successfully infected cells were selected using 1 μg/mL puromycin.

### Protein extraction and Western blot analysis

Cells were grown in CTR or STS media with or without holo-transferrin or hemin and then treated with vitamin C (350 µM) or vehicle for 3 h. Next, cells were washed twice in ice-cold PBS and lysates were prepared in RIPA lysis buffer (50 mM Tris HCl pH 7.4, 150 mM NaCl, 1% NP-40, 0.25% deoxycholic acid, 1 mM EDTA) supplemented with protease and phosphatase inhibitors (protease inhibitor cocktail set III EDTA-free, Calbiochem, Cat. #: S39134; PhosStop, Roche). Samples were sonicated (Bioruptor Plus, Diogenode) and centrifuged at 16.1 × *g* for 30 min at 4 °C. Tumor tissues were collected and snap frozen in liquid nitrogen immediately after mice were sacrificed, and stored in −80 °C until use. For protein extraction, tumors were homogenized with Tissue lyser II (Qiagen) in RIPA buffer supplemented with protease and phosphatase inhibitors and then ultra-centrifuged (122,245  × *g* using a MLA-130 Beckman rotor) for 1 h. Protein concentrations were determined by BCA assay (Thermo Fisher Scientific, Cat. #: 23225). Proteins were resolved by SDS page and analyzed by immunoblotting using antibodies for HO-1 (1:1000, Enzo Life Science, Cat. #: ADI-SPA894), FTH1 (1:1000, Cell Signaling, Cat. #: 3998), H2AX (1:4000, Abcam, Cat. #: ab11175), phospho serine 139 H2AX (1:5000, Merck Millipore, Cat. #: 05636), VINCULIN (1:10,000, Sigma-Aldrich, Cat. #: V9131), β-ACTIN (1:3000, Sigma, Cat. #: A2066). Immunostained bands were detected under a ChemiDoc imaging system (Biorad) using the chemiluminescent method (super signal west PICO and super signal west DURA, Thermo Fisher, Cat. #: 34577, Cat. #: 34075). Bands intensity was quantified with NIH Image J software (version 1.50i). Uncropped original blots are shown in Supplementary Figure 11.

### RNA extraction, RT-PCR, and qRT-PCR

Total RNA was isolated using the miRNeasy Mini Kit (QIAGEN, #217004) according to the manufacturer’s instructions. Briefly, 1 μg of purified RNA was retro-transcribed by using SuperScript Vilo cDNA synthesis kit (Invitrogen, #11754050). Resulting cDNA (1/20 v/v) was analyzed by real-time polymerase reaction (RT-PCR) using QuantStudio 12 K flex Real Time PCR system (Thermo Fisher). Human target gene primers for heme-oxygenase-1 (HMOX1:Hs01110250_m1, ThermoFisher Scientific) were utilized. Target transcript levels were normalized to those of a reference gene (GAPDH: hs99999905_m1, ThermoFisher Scientific). To confirm *KRAS* overexpression in HT29 cell line mRNA levels were detected using SYBR Green GoTaq® qPCR Master Mix (Promega) according to the manufacturer’s protocol. The sequences of the used primer were CGGGAAATCGTGCGTGACATTAAG (ACTIN FW), TGATCTCCTTCTGCATCCTGTCGG (ACTIN REV), GGGGAGGGCTTTCTTTGTGTA (KRAS FW) and GTCCTGAGCCTGTTTTGTGTC (KRAS REV). Gene expression was normalized to housekeeping gene expression (β-Actin). Comparisons in gene expression were calculated using the 2^−ΔΔCt^ method.

### Mouse models

The animals were housed under specific pathogen-free conditions at 22 ± 2 °C with 55 ± 10% relative humidity and with 12 h day/light cycles. All experiments were performed in accordance with the guidelines established in the Principles of Laboratory Animal Care (directive 86/609/EEC), were approved by the Italian Ministry of Health, and were performed under the supervision of the institutional organism for animal welfare (Cogentech OPBA). For xenograft experiments, 8-week-old female NOD scid gamma (NSG, Charles River) were subcutaneously injected with 2 × 10^6^ HCT116 cells (NCI 60 panel) resuspended in 100 μL of PBS. For syngeneic model, 8-week-old female BALB/cOlaHsd mice (Envigo) were subcutaneously injected with 3 × 10^5^ CT26 (ATCC) cells resuspended in 100 μL of PBS. When tumors were palpable (7 days after inoculation), mice were randomly divided in the different experimental groups. Body weights were recorded daily, and tumor volumes were measured every 2–3 days by a digital vernier caliper according to the following equation: tumor volume (mm^3^) = (length × width^2^) × 0.5, where the length and width are expressed in millimeters. For the orthotopic model, the animal protocol was approved by the Institutional Animal Care and Use Committee (IACUC) of the University of Southern California. Female BALB/c mice (8-week old, strain 000651-Jackson Laboratory) were anesthetized with isoflurane anaesthesia. Mice then received a gentle anal dilation using blunt-tipped forceps at the anal opening. A 29-gauge syringe was used to inject 2.5 × 10^4^ CT26-luc cells (SC061-LG GenTarget Inc), suspended in saline, submucosally into the distal, posterior rectum. Seven days later, mice were randomly divided in the different experimental groups. Twenty-one days post injections mouse imaging was performed using the Xenogen IVIS-200 System. Mice were anesthetized by isoflurane anaesthesia and luciferin (50 mg/kg body weight) was administered via intra-peritoneal injections and animals were subjected to Bioluminescence Imaging (BLI) at the USC Small Animal Imaging Center. Bioluminescent imaging data were analysed by Living Image 4.3.1. At the end of the experiments, mice were euthanized by using CO_2_.

### Animal diets and treatments

Mice were fed ad libitum with irradiated VRFI (P) diet (Charles River) containing 3.89 kcal/g of gross energy. Our FMD diet is based on a nutritional screen that identified ingredients that allow nourishment during periods of low calorie consumption^23^. The FMD diet consists of two different components designated as day 1 diet and days 2–4 diet. Day 1 diet contains 7.67 kJ/g (provided 50% of normal daily intake; 0.46 kJ/g protein, 2.2 kJ/g carbohydrate, 5.00 kJ/g fat); the day 2–4 diet contains 1.48 kJ/g (provided at 10% of normal daily intake; 0.01 kJ/g protein/fat,1.47 kJ/g carbohydrates). Before FMD diet was supplied, mice were transferred in fresh cages to avoid residual chow feeding and coprophagy. Mouse weight was monitored daily and during FMD cycle, weight loss did not exceed 20%.

For experiments on tumor growth, mice were fed with standard rodent diet or underwent FMD cycles (3 days each). In experiments with chemotherapy, the second FMD cycle was reduced to 2 days because the faster rate of body weight loss. Before FMD cycle was repeated, mice completely recovered their original bodyweight.

For vitamin C experiments, mice undergoing standard feeding or at the last day of the first FMD cycle started to be treated with vitamin C (4 g/kg in saline) via intraperitoneal injection twice a day, every day until the end of the experiment. At least 6–8 h have elapsed between the two administrations in each day.

For chemotherapy experiments, mice undergoing standard feeding or at the second day of the first FMD cycle started to be treated with vitamin C twice a day (until day 36), and during the last day of each FMD cycle (24 h before refeeding), mice were treated with oxaliplatin (10 mg/kg) via intraperitoneal injection once every 15 days for NSG and 11 days for Balb/c mice (according to the mouse body weight recovery). When mice were treated with chemotherapy, vitamin C injection was skipped. At least 8–9 h have elapsed between vitamin C and oxaliplatin administrations.

### In vivo iron-bound transferrin measurement

For iron-bound transferrin measurement, mouse blood was collected from the heart of mice sacrificed at the end of second FMD cycle and 24 h post-refeeding. Blood was incubated at room temperature (25 °C) for at least 30 min to clot and then centrifuged for 15 min at 2000  × *g* (4 °C). Collected serum was aliquoted and stored at −80 °C. Iron-bound transferrin was measured by Serum Iron Assay kit (Biovision, #K392) according to manufacturer protocol.

### TCGA patient’s analysis

TCGA-COAD patient information, including gender, mutational status and clinical data, could be retrieved through GDC Data Portal (https://portal.gdc.cancer.gov/projects/TCGA-COAD). Sequencing of human subjects’ tissue was performed by TCGA consortium members under a series of locally approved Institutional Review Board (IRB) protocols as described in (Cancer Genome Atlas Network (2012)). Informed consent was obtained from all human participants.

Molecular data and clinical information including *KRAS* status and, survival and RNA-seq FTH1 mRNA expression data in TCGA-COAD (*n* = 266) samples were collected from GDC Data Portal (https://portal.gdc.cancer.gov) and TSVdb^39^ respectively. Patients with undefined survival time or without available RNA-seq data were excluded, only primary solid tumors were considered. *KRAS*-mutant tumors were selected according to the presence of a predicted deleterious mutation (DH; SIFT value <0.05). Patients were stratified according to the median tumor *FTH1* mRNA expression level.

### Statistical analysis

GraphPad Prism v8.0.2 (159) was used for the analysis of the data and graphic representations. Comparisons between groups were performed with two-sided unpaired Student’s *t*-test. One-way ANOVA analysis was used for comparison among multiple groups for mouse experiments. One-way ANOVA analysis was followed by Tukey’s test post analysis. *P* values ≤ 0.05 were considered significant. For in vivo experiments, we evaluated the sample size by G.Power software considering a multifactorial variance analysis. We obtained that *n* = 10 mice per group can reach a power of 0.9 (subject to alpha = 0.05). Outlier presence was checked and removed according to ROUT method (ROUT = 1%). A confidence interval of 95% (*P* values ≤ 0.05) were considered significant. For survival analysis, the Log-rank (Mantel-Cox) test was performed. For clinical data analysis, Gehan–Breslow–Wilcoxon test was utilized. All data are represented as mean ± SEM of at least three independent experiments.

### Reporting summary

Further information on experimental design is available in the Nature Research Reporting Summary linked to this paper.

## Supplementary information

Supplementary Information

Reporting Summary

## Data Availability

All patient’s survival data that support the findings of this study are available in GDC Data Portal (https://portal.gdc.cancer.gov/projects/TCGA-COAD). All other data generated or analysed during this study are included in this published article (and its supplementary information files).
