# Supplementary material for: Synergistic effect of fasting-mimicking diet and vitamin C against KRAS mutated cancers
Source: Nat Commun. 2020 May 11;11:2332. doi: 10.1038/s41467-020-16243-3 (PMC7214421; doi:10.1038/s41467-020-16243-3)

Supplementary Information

Synergistic effect of Fasting-mimicking diet and vitamin C against *KRAS* mutated cancers

Di Tano et al.

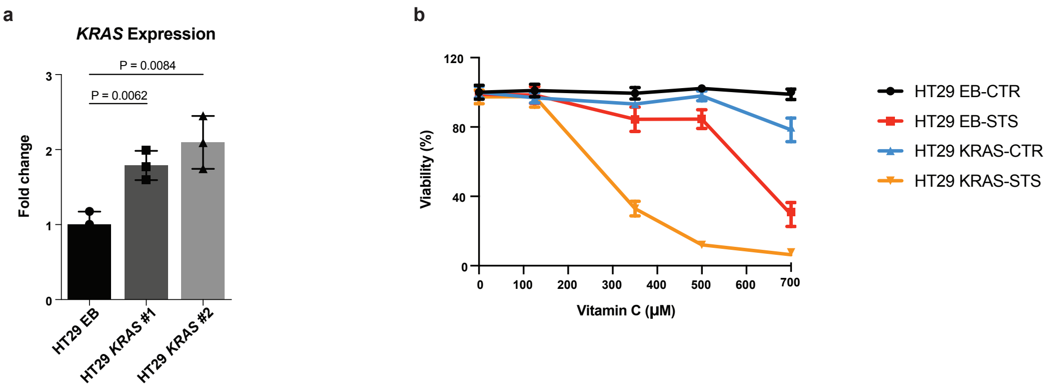

Supplementary Figure 1. *KRAS* mutant CRC cells are more susceptible to STS + Vitamin C toxic effect than their isogenic wild type counterpart. (a) HT29 cells (n=3 independent samples) infected with empty backbone (EB) or *KRAS* plasmid (2 independent infections are indicated as #1, #2) are checked for *KRAS* expression by qPCR. P values were determined by two-sided unpaired *t*-test (**b**) Viability of HT29 EB/KRAS grown in CTR/STS medium with different concentrations of vitamin C is measured by CellTiter96 AQ_{ueous} cell proliferation assay (n=12 technical replicates in CTR and STS groups, n=6 technical replicates in vitamin C treated groups). Data are represented as mean ± SD.

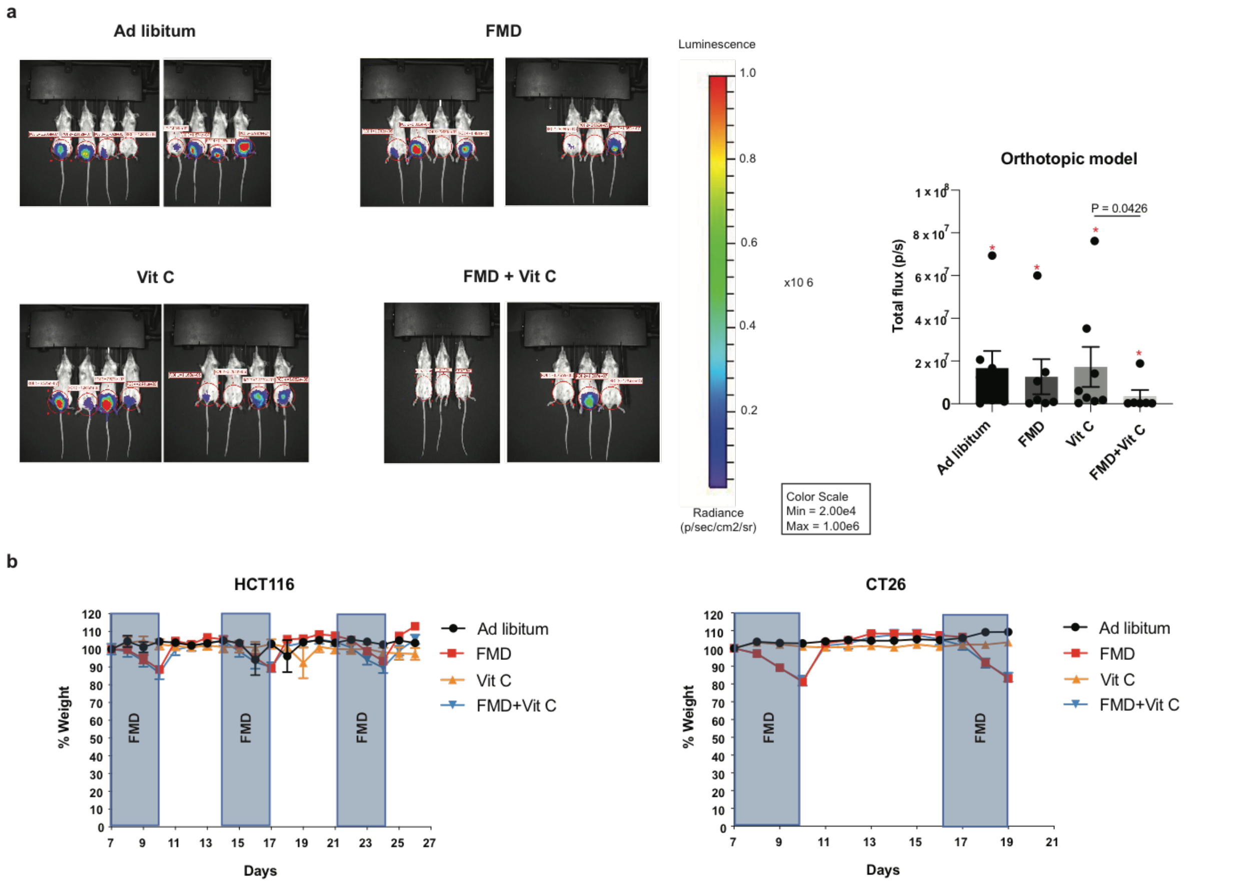

Supplementary Figure 2. FMD + Vitamin C treatment is effective, safe and well tolerated in CRC models. (a) CT26-luc cells were injected submucosally in the distal posterior rectum (n=8 in Ad libitum and Vit C groups, n=7 in FMD group and n=6 in FMD + Vit C group). Bioluminescence picture (left side) are presented. Total photon effluxes over tumor regions of all mice included outliers are presented. Outliers were identified according to ROUT method and marked with red asterisk. Two-sided non-parametric t-test with Mann-Whitney correction was used (P= 0.0426). Data are represented as mean ± SEM.

(b) Bodyweight of NGS mice (n=10 in FMD and FMD + Vit C groups, n=6 in Ad libitum group, n=7 in Vit C group) bearing HCT116 derived tumors (left) and Balb/c OlaHsd mice (n=12 in Ad libitum group; n=10 in FMD, Vit C and FMD + Vit C) bearing CT26-derived tumors (right) undergoing 3-days FMD alone or in combination with vitamin C (Vit C; 4g/kg twice a day, i.p.). Bodyweight was recorded daily and it is indicated as percentage of weight at day 7. Data are represented as mean ± SEM.

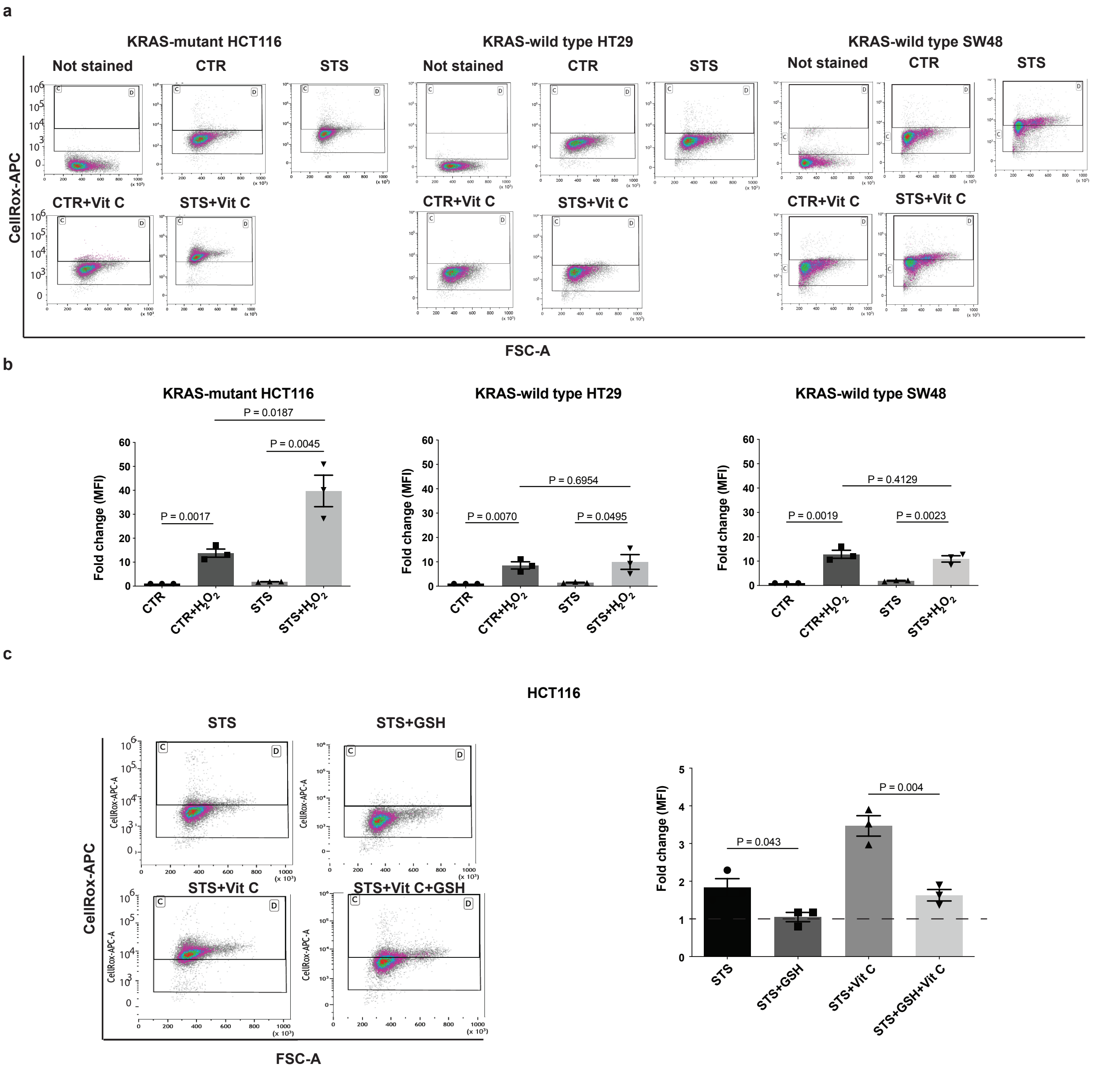

Supplementary Figure 3. STS enhances vitamin C/H_2O_2-dependent ROS increase in *KRAS*-mutant cancer cells and GSH reverts this effect. CellRox-Deep-Red detection by flow-cytometry (MFI: Median fluorescence intensity) of: **(a)** HCT116 (n=7), HT29 (n=3) and SW48 (n=3) cells grown in CTR or STS medium and treated with or without vitamin C (Vit C; 1 mM). Representative dot plots are shown; **(b)** HCT116, HT29 and SW48 cells grown in CTR or STS medium and treated with or without H_2O_2 (200 µM) (n=3). Quantifications are shown. P values were determined by two-sided unpaired *t*-test; (**c**) Dot plot (left) and quantification relative to CTR cells (right) of HCT116 grown in STS condition and treated with or without glutathione (GSH, 5 mM) one hour prior to vitamin C treatment (n=3). P values were determined by two-sided unpaired *t*-test. All data are represented as mean ± SEM, n=independent experiments.

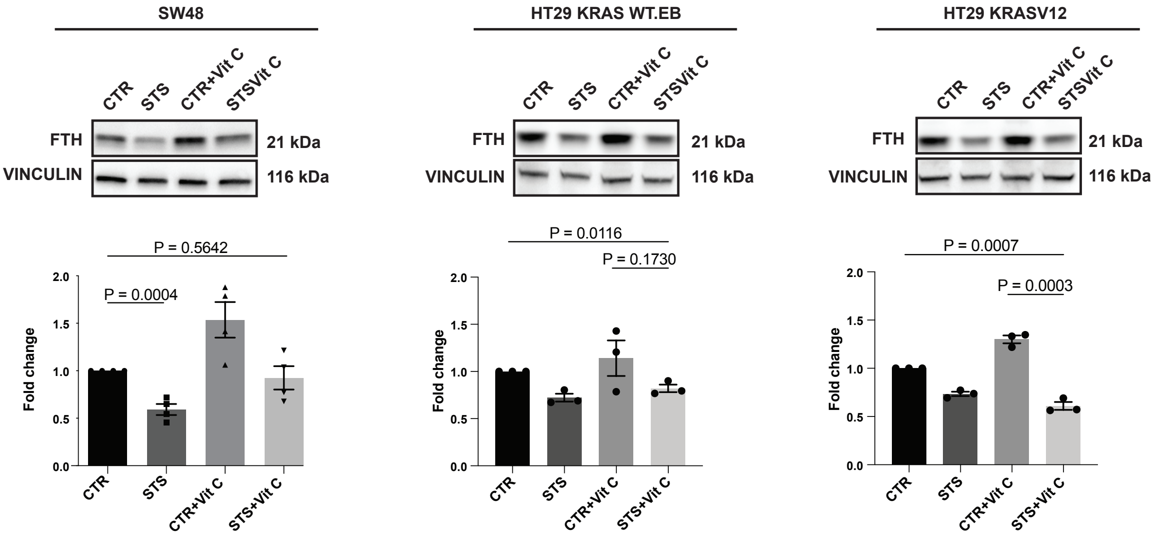

Supplementary Figure 4. FTH is differentially regulated upon STS + Vitamin C according to *KRAS* mutational status. Detection of FTH by western blot in SW48 cancer cells (*KRAS* wild type, n=4), in HT29 (*KRAS* wild type) transduced with empty backbone (EB, n=3) or expressing the mutant form of *KRAS* (KRASV12, n=3) grown in CTR or STS medium for 24 hours and then treated with vitamin C (350µM) for 3 hours (representative blots in the upper panels). Quantification in the bottom panels. VINCULIN as loading control. All P values were determined by two-sided unpaired *t*-test. All data are represented as mean ± SEM, n=independent experiments.

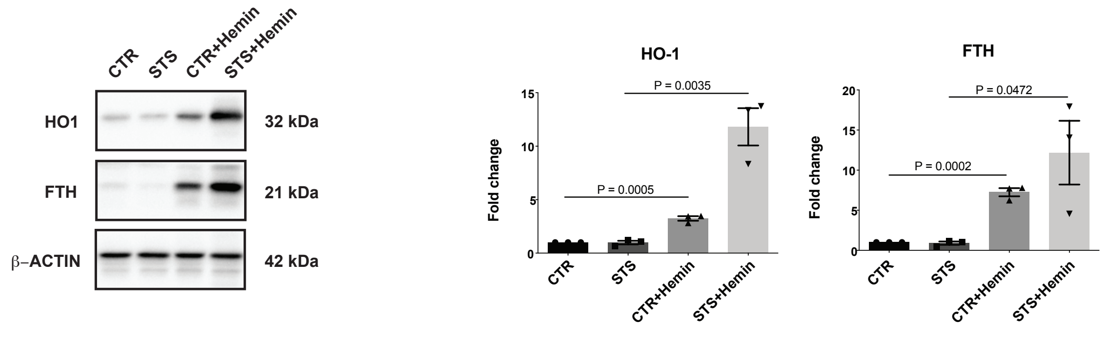

Supplementary Figure 5. Hemin up-regulates HO-1 and FTH protein expression level. HCT116 were grown in CTR or STS condition for a total of 24 hours. At 12 hours, cells were treated with the HO-1 activator hemin (20 µM) for the next 12 hours. HO-1 and FTH protein expression level were measured by western blotting; β-ACTIN as loading control. Representative bands of three independent experiments are shown (quantifications on the right). P values were determined by two-sided unpaired *t*-test. Data are represented as mean ± SEM.

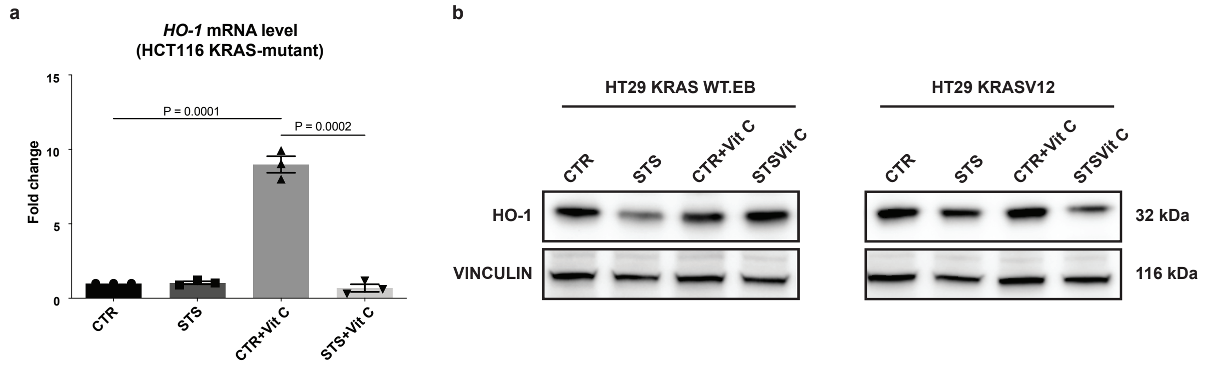

Supplementary Figure 6. STS and Vitamin C differentially regulate HO-1 expression level in *KRAS*-wild type and *KRAS*-mutant CRC cells. (a) qPCR of *HO-1* mRNA level, relative to *GAPDH*, in HCT116 cells (n=3). P values were determined by two-sided unpaired *t*-test. Data are represented as mean ± SEM. (**b**) Detection of HO-1 protein level by western blot in HT29 (*KRAS*-wild type) cells transduced with empty backbone (EB) or with KRASV12 vector (*KRAS*-mutant). VINCULIN as loading control. Representative bands are shown (n=3), n=independent experiments.

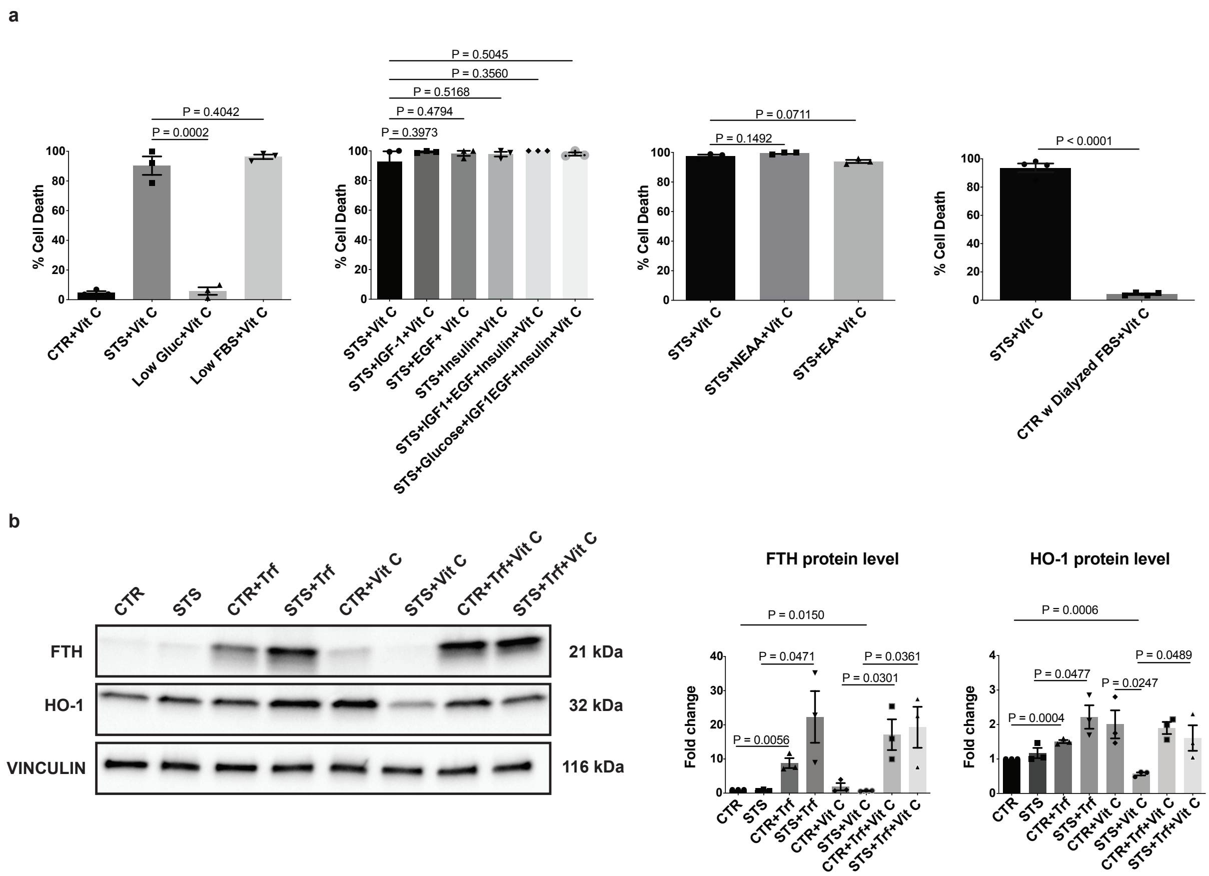

Supplementary Figure 7. STS + vitamin C toxicity is dependent on serum iron bound transferrin, which reverses HO-1/FTH down-regulation. (**a**) Viability of HCT116 grown in the indicated growing media for 48 hours with or without vitamin C (350μM; n=4 in STS + Vit C/CTR w Dialyzed FBS + Vit C graph, n=3 in all other graphs). CTR=control medium; STS=starvation medium, Low Gluc=CTR with 0.5 g/L glucose, Low FBS=CTR with 1% serum, IGF-1 (Insulin Growth Factor-1; 250 ng/Ml), EGF (Epidermal Growth Factor; 200 ng/mL), Insulin (200 ng/mL), Glucose (1 g/L), NEAA (non-essential amino acids; 1 mM), EAA (essential amino acids, 2X), CTR w Dialyzed FBS=CTR with 10% dialyzed serum). P values were determined by two-sided unpaired *t*-test. Exact P value= 0.0000001 (STS+Vit C vs CTR w dialyzed FBS+Vit C). **(b)** HCT116 cells were grown in CTR or STS medium, in presence or absence of holo-transferrin (0.3 mg/ml) for 24 hours before treatment with vitamin C (350 μM for 3 hours). FTH and HO-1 protein expression levels were detected by western blotting (n=3). VINCULIN as loading control. Representative blot on the left side, quantification on the right. P values were determined by two-sided unpaired *t*-test. All data are represented as mean ± SEM, n=independent experiments.

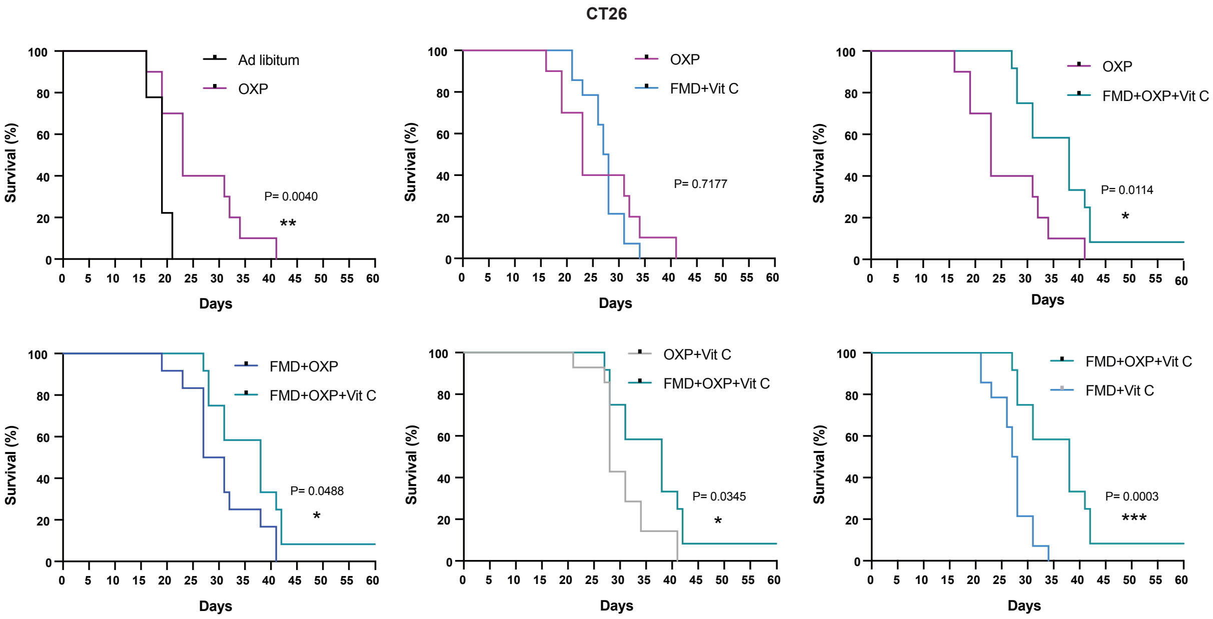

Supplementary Figure 8. FMD, vitamin C and oxaliplatin triple treatment extends mouse survival. BALB/c mice were subcutaneously injected with CT26 cells. Mice were fed *ad libitum* or subjected to FMD cycles, and treated with or without vitamin C or oxaliplatin. Survival curves and statistical analysis are shown (Log-rank (Mantel-Cox) test was performed) (n=9 mice in Ad libitum group, n=10 mice in OXP group, n=12 mice in FMD + OXP and FMD + OXP + Vit C, n=14 mice in OXP + Vit C and FMD + Vit C groups).

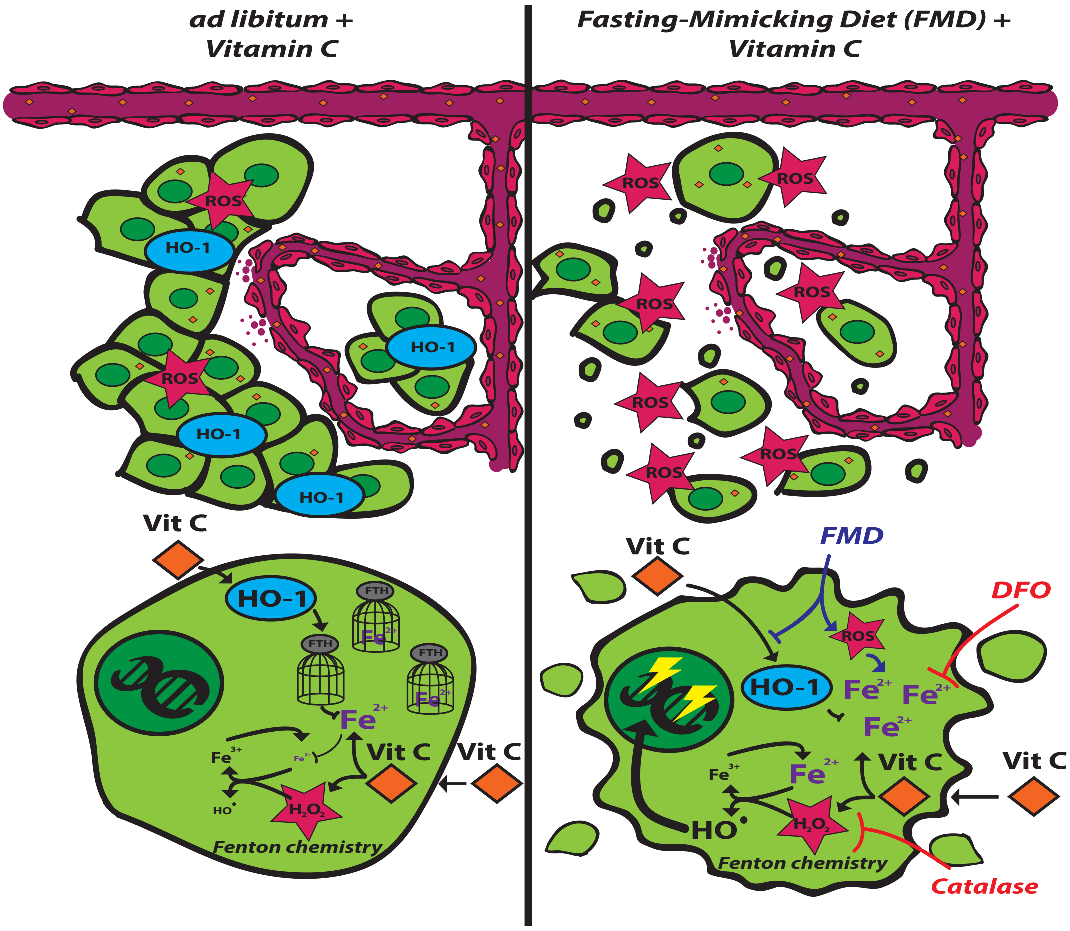

Supplementary Figure 9. Working model of FMD-mediated sensitization to vitamin C. Vitamin C oxidation generates H_2O_2 that, by reacting with Fe^{2+} (Fenton chemistry), produces $HO^{•}$. Vitamin C-mediated up-regulation of HO-1 induces ferritin (FTH), thus limiting labile iron pool (LIP) and consequently the pro-oxidant chemistry responsible for vitamin C toxicity (left). FMD is able to revert the HO-1 up-regulation induced by vitamin C. By doing this, FMD expands Fe^{2+} pool possibly through FTH down-regulation. The increase in Fe^{2+}, together with the FMD-induced boost in ROS levels possibly exacerbate Fenton chemistry leading to DNA damage (yellow bolts) and cell death. Catalase, by scavenging H_2O_2, and DFO, by chelating iron, inhibit Fenton reaction and prevent cell death (right).

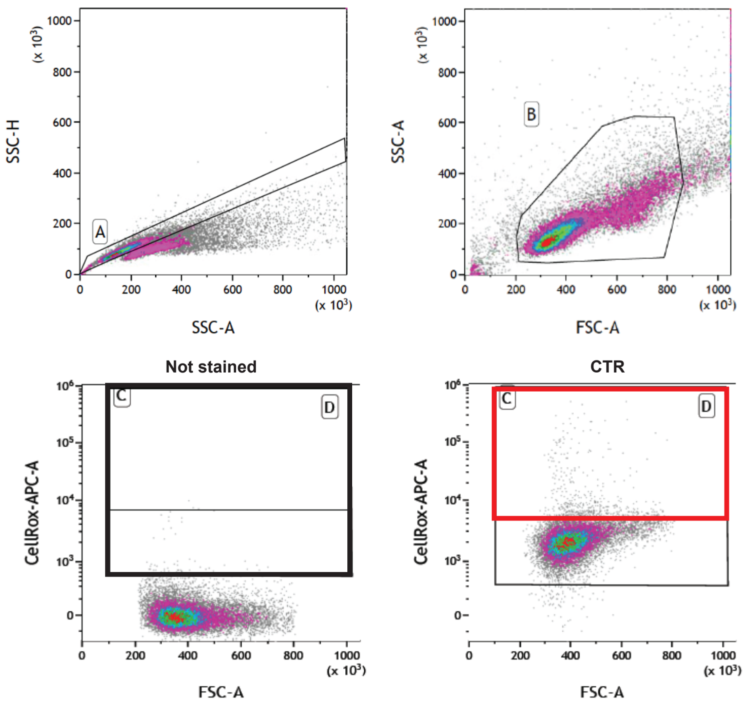

Supplementary Figure 10. Gating strategy for ROS detection by FACS. Cells were first gated based on the size (FSC) and on density and granularity of the cells (SSC) as shown in the scheme (panel above) to exclude debris, doublets and select only live fraction. A first gate (black square, panel below) was applied above the not-stained negative control cells to identified stained-positive cells. A second gate (red square) was applied above the fluorescence level of the control condition (CTR). The fluorescence intensities of treated samples were compared to the fluorescence intensity of control sample.

Supplementary Figure 11. Uncropped original blots

Cropped regions shown in indicated main figures are marked with red boxes.

Figure 2a

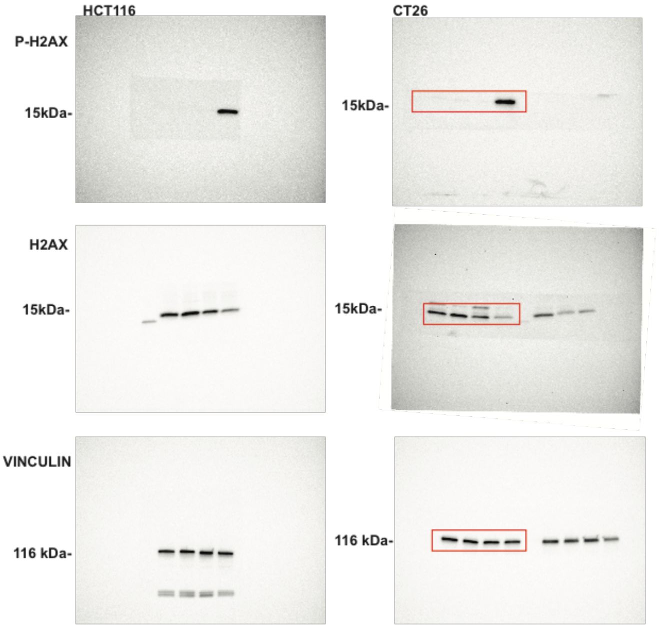

Figure 3b,c

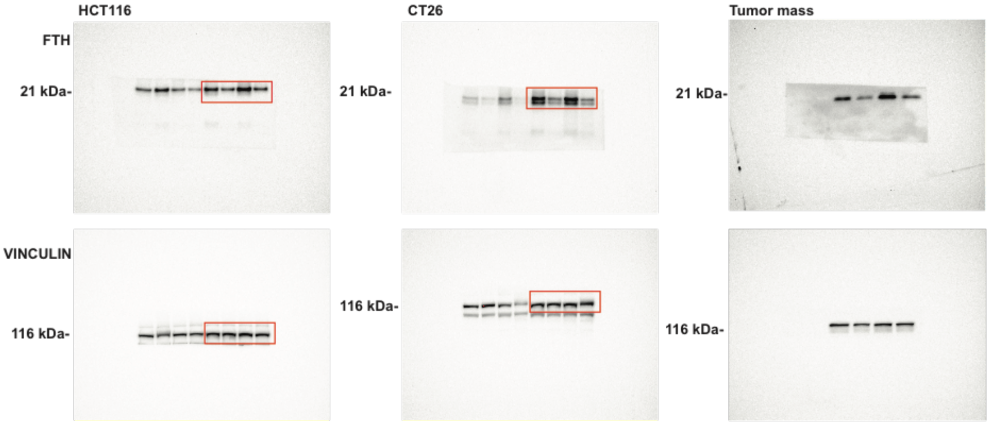

Figure 4a,b

HCT116 CT26 Tumor mass

HO-1

32 kDa- 32 kDa- 32 kDa

VINCULIN

116 kDa- 116 kDa- 116 kDa-

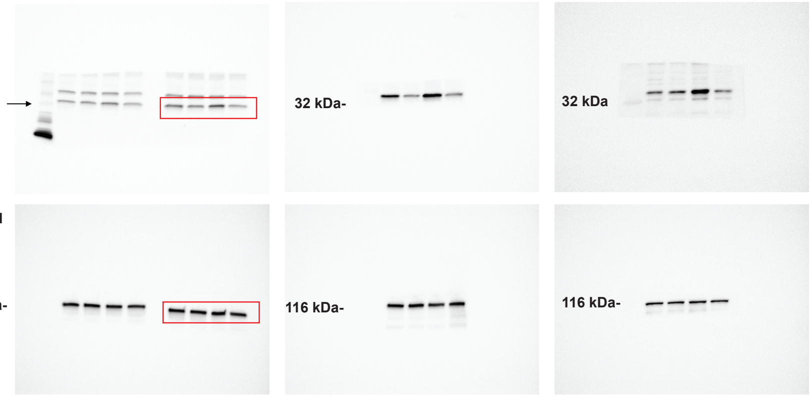

SW48

HO-1 VINCULIN

32 kDa- 116 kDa-

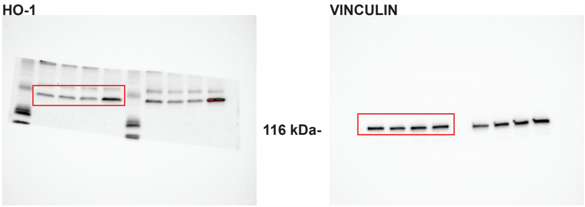

Figure 4e

HO-1

32 kDa-

β-ACTIN

42 kDa-

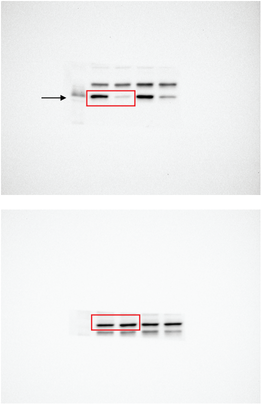

Supplementary Figure 4

SW48 HT29 WT.EB HT29 KRASV12

FTH

21 kDa- 21 kDa-

VINCULIN

116 kDa- 116 kDa-

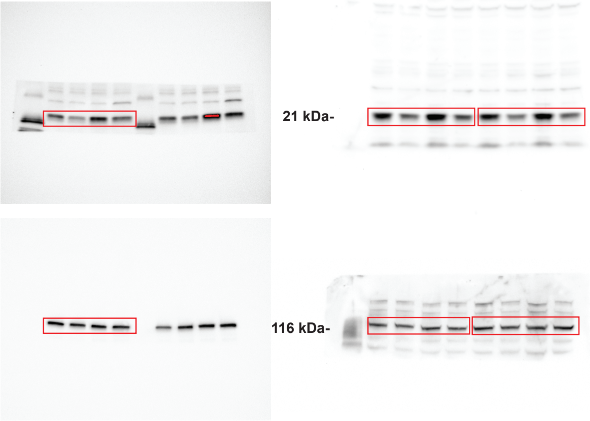

Supplementary Figure 5

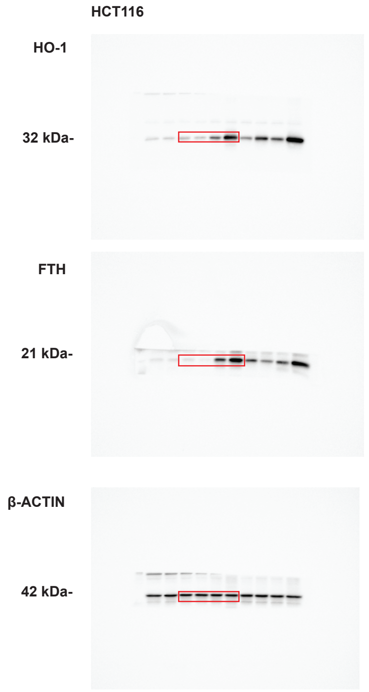

Supplementary Figure 6

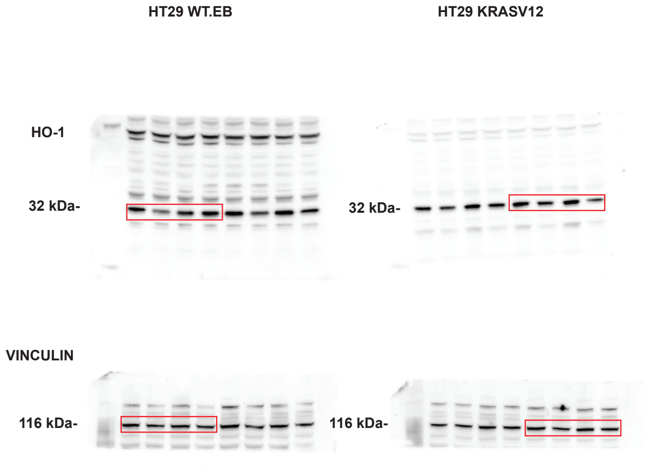

Supplementary Figure 7

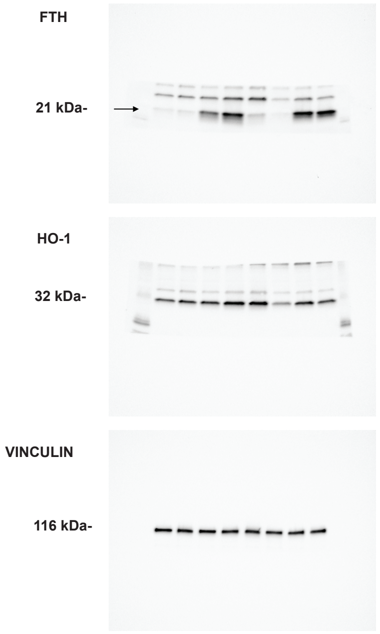

Supplement: Supplementary file 1 — Supplementary Information [file 41467_2020_16243_MOESM1_ESM.pdf]
