# Supplementary material for: Synergistic effect of fasting-mimicking diet and vitamin C against KRAS mutated cancers
Source: Nat Commun. 2020 May 11;11:2332. doi: 10.1038/s41467-020-16243-3 (PMC7214421; doi:10.1038/s41467-020-16243-3)
Supplement: Supplementary file 2 — Reporting Summary [file 41467_2020_16243_MOESM2_ESM.pdf]

nature research

Corresponding author(s): VALTER D. LONGO

Last updated by author(s): Apr 6, 2020

Reporting Summary

Nature Research wishes to improve the reproducibility of the work that we publish. This form provides structure for consistency and transparency in reporting. For further information on Nature Research policies, see Authors & Referees and the Editorial Policy Checklist .

Statistics

For all statistical analyses, confirm that the following items are present in the figure legend, table legend, main text, or Methods section.

n/a	Confirmed	
☐	☒	The exact sample size (*n*) for each experimental group/condition, given as a discrete number and unit of measurement
☐	☒	A statement on whether measurements were taken from distinct samples or whether the same sample was measured repeatedly
☐	☒	The statistical test(s) used AND whether they are one- or two-sided<br>*Only common tests should be described solely by name; describe more complex techniques in the Methods section.*
☒	☐	A description of all covariates tested
☐	☒	A description of any assumptions or corrections, such as tests of normality and adjustment for multiple comparisons
☐	☒	A full description of the statistical parameters including central tendency (e.g. means) or other basic estimates (e.g. regression coefficient) AND variation (e.g. standard deviation) or associated estimates of uncertainty (e.g. confidence intervals)
☐	☒	For null hypothesis testing, the test statistic (e.g. *F*, *t*, *r*) with confidence intervals, effect sizes, degrees of freedom and *P* value noted<br>*Give P values as exact values whenever suitable.*
☒	☐	For Bayesian analysis, information on the choice of priors and Markov chain Monte Carlo settings
☒	☐	For hierarchical and complex designs, identification of the appropriate level for tests and full reporting of outcomes
☒	☐	Estimates of effect sizes (e.g. Cohen's *d*, Pearson's *r*), indicating how they were calculated

Our web collection on statistics for biologists contains articles on many of the points above.

Software and code

Policy information about availability of computer code

Data collection	-FACS analysis: samples were acquired by Attune NxT flow cytometer.<br>-Western blot: immunostained bands were detected under a ChemiDoc imaging system (Biorad)<br>-Real time PCR: QuantStudio 12K flex Software v1.3 (Thermo Fisher)<br>-Viability assay: Muse cell analyzer (Merck Millipore)<br>-Colorimetric assay(absorbance): Infinite M200 TECAN<br>-In vivo bioluminescent imaging: Xenogen IVIS 200
Data analysis	-For FACS analysis data were processed by Kaluza analysis software (Beckman coulter, version 2.0).<br>-For western blot bands intensity was quantified with NIH Image J software (version 1.50i).<br>- For Real time PCR 12K Flex software v1.3 was used.<br>-For in vivo bioluminescent imaging data were analyzed by Living Image 4.3.1<br>-For statistical analysis and graphic representation: GraphPad Prism v8.0.2 (159)

For manuscripts utilizing custom algorithms or software that are central to the research but not yet described in published literature, software must be made available to editors/reviewers. We strongly encourage code deposition in a community repository (e.g. GitHub). See the Nature Research guidelines for submitting code & software for further information.

Data

Policy information about availability of data

All manuscripts must include a data availability statement. This statement should provide the following information, where applicable:

- Accession codes, unique identifiers, or web links for publicly available datasets
- A list of figures that have associated raw data
- A description of any restrictions on data availability

All patient’s survival data that support the findings of this study are available in GDC Data Portal (https://portal.gdc.cancer.gov/projects/TCGA-COAD). All other data

generated or analysed during this study are included in this published article (and its supplementary information files).

Field-specific reporting

Please select the one below that is the best fit for your research. If you are not sure, read the appropriate sections before making your selection.

- [x] Life sciences
- [ ] Behavioural & social sciences
- [ ] Ecological, evolutionary & environmental sciences

For a reference copy of the document with all sections, see nature.com/documents/nr-reporting-summary-flat.pdf

Life sciences study design

All studies must disclose on these points even when the disclosure is negative.

Sample size	For in vivo experiments, we evaluated the sample size by G.Power software considering a multifactorial variance analysis. We obtained that n=10 mice per group can reach a power of 0.9 (subject to alpha= 0.05). The rationale for this was to reduce to a minimum the number of animals used, according to the 3R principle for animal care. For all other experiments no statistical methods were used to predetermine sample size. Sample sizes were estimated on the basis of previous experiments conducted in our laboratory providing a sufficient numerosity for each group to yield a two-sided statistical test that can reach a power of 0.9 (subject to alpha= 0.05).
Data exclusions	No data were excluded for pre-established criteria. For all the experiments, the Robust regression and Outlier removal method (ROUT=1%) was performed. In the orthotopic in vivo experiment, 1 outlier per group was identified and data were analyzed both with and without outliers.
Replication	We confirm that all experiments were reproduced at least three independent times. To verify their reproducibility the experiments were repeated using different lots of each reagents, different stocks of cell lines and some experiments were also repeated and confirmed by other operators, obtaining successful replication of the results.
Randomization	Samples and mice were randomly assigned to the different experimental groups.
Blinding	For in vivo dietary studies blinding to group allocation and analysis was not possible as the same operators performed dietary, pharmacological treatment and downstream analyses. For all the in vitro experiments, during the experimental procedure blinding to group allocation and analysis was not possible since cells need to be treated with different reagents and cultured in different growing media (CTR and STS).

Reporting for specific materials, systems and methods

We require information from authors about some types of materials, experimental systems and methods used in many studies. Here, indicate whether each material, system or method listed is relevant to your study. If you are not sure if a list item applies to your research, read the appropriate section before selecting a response.

Materials & experimental systems

n/a	Involved in the study	
[ ]	[x]	Antibodies
[ ]	[x]	Eukaryotic cell lines
[x]	[ ]	Palaeontology
[ ]	[x]	Animals and other organisms
[ ]	[x]	Human research participants
[x]	[ ]	Clinical data

Methods

n/a	Involved in the study	
[x]	[ ]	ChIP-seq
[ ]	[x]	Flow cytometry
[x]	[ ]	MRI-based neuroimaging

Antibodies

Antibodies used	HO-1 (1:1000; Enzo Life Science, Cat. #: ADI-SPA894), FTH (1:1000; Cell Signaling, Cat. #: 3998), H2AX (1:4000; Abcam, Cat. #: ab11175), phospho serine 139 H2AX (1:5000, Merck Millipore, Cat. #: 05636), Vinculin (1:10000, Sigma-Aldrich, Cat. #: V9131), β-actin (1:2000; Sigma, Cat. #: A2066) (western blotting).
Validation	All antibodies are commercially available and were tested for species and application as described in each manufacturer's protocol:<br>-HO-1 (1:1000; Enzo Life Science, Cat. #: ADI-SPA894). Rabbit polyclonal. Validated to react in Human, Mouse, Rat,Dog, Guinea pig, Hamster, Monkey, Rabbit, Sheep; Validated for immunohystochemistry (IHC) and western blot (WB). Tested in HO-1 (human), (recombinant) (Product No. ADI-SPP-732), Human Liver Microsomes, Mouse Liver Microsomes,Rat Liver Microsomes. REF: Globular adiponectin protects hepatocytes from tunicamycin-induced cell death via modulation of the inflammasome and heme oxygenase-1 induction: A. Khakurel & P.H. Park; Pharmacol. Res. 128, 231 (2018).<br><br>-FTH (1:1000; Cell Signaling, Cat. #: 3998). Rabbit polyclonal. Validated to react in Human, Mouse, Rat, Monkey. Validated for

western blot (WB). Tested in HT29 and Hela cell extracts REF: Sun, Y., Li, C., et al. (2019), 'Ferritinophagic Flux Activation in CT26 Cells Contributed to EMT Inhibition Induced by a Novel Iron Chelator, DpdtpA.

-H2AX (1:4000; Abcam, Cat. #: ab11175). Rabbit polyclonal. Validated to react in Human, Mouse, Rat, Monkey. Validated for immunohistochemistry-paraffin/frozen section (IHC-P/Fr), western blot (WB), chromatin immunoprecipitation (ChIP), immunoprecipitation (IP), immunocytochemistry/immunofluorescence (ICC/IF). Tested in with human HEK293, human G-361 and mouse embryonic fibroblast cells. REF: Li L et al. SIRT7 is a histone desuccinylase that functionally links to chromatin compaction and genome stability. Nat Commun 7:12235 (2016).

-phospho serine 139 H2AX (1:5000, Merck Millipore, Cat. #: 05636, clone JBW301). Mouse monoclonal. Validated to react in vertebrates. Validated for western blot (WB), immunocytochemistry (ICC), immunohistochemistry (IHC), chromatin immunoprecipitation (ChIP), immunofluorescence (IF). Control: UV-treated 293 cell extracts, UV-treated HeLa cell extracts or breast cancer tissue. REF: HMGB1 facilitates repair of mitochondrial DNA damage and extends the lifespan of mutant ataxin-1 knock-in mice.Ito, H; Fujita, K; Tagawa, K; Chen, X; Homma, H; Sasabe, T; Shimizu, J; Shimizu, S; Tamura, T; Muramatsu, S; Okazawa, HEMBO molecular medicine 7 78-101 2015.

-VINCULIN (1:10000, Sigma-Aldrich, Cat. #: V9131).Mouse monoclonal. Validated to react in bovine, canine, mouse, rat, turkey, human, chicken, frog. Validated for western blot (WB), immunohistochemistry (IHC), immunofluorescence (IF). Tested in HeLa COS7, NIH-3T3, RAT2, CHO, MDBK, MDCK cell lysates. REF: Somatic inactivation of the PHD2 prolyl hydroxylase causes polycythemia and congestive heart failure Minamishima YA, et al. Blood 111(6), 3236-3244, (2008).

- β-ACTIN (1:200,Sigma, Cat. # :A2066). Rabbit polyclonal. Validated to react in wide range, human, chicken, amoeba, slime mold, vertebrates. Validated for immunohistochemistry (formalin-fixed, paraffin-embedded sections), indirect immunofluorescence, western blot. Tested in JURKAT, COS7, P19, PC-12, RAT2, CHO, MDBK, MDCK cell lysates. Actin detected by a rabbit anti-actin antibody was used as a loading control for MCF7 cell lysates according to manufacturer's protocol. REF: Sarkar S, Davies JE, Huang Z, Tunnacliffe A, Rubinsztein DC. Trehalose, a novel mTOR-independent autophagy enhancer, accelerates the clearance of mutant huntingtin and alpha-synuclein. J Biol Chem. 2007;282(8):5641–5652.

Eukaryotic cell lines

Policy information about cell lines

Cell line source(s)	HCT116, HT29, NCI-H23 and PC-3 cells were obtained from NCI 60 panel; CT26.WT, CCD-84CoN were purchased from ATCC; DLD1 cell line was purchased from DSMZ; NCI-H727, PANC-1, Cov362 and SW48 cells were purchased from ECACC, and SW48 KRAS G12D/+ were purchased from Horizon Discovery. CT26-luc cells were purchased from GenTarget Inc (SC061-LG).
Authentication	Cell lines were authenticated by STR profiling
Mycoplasma contamination	All cell lines were routinely tested for mycoplasma contamination and results were negative.
Commonly misidentified lines (See ICLAC register)	None

Animals and other organisms

Policy information about studies involving animals; ARRIVE guidelines recommended for reporting animal research

Laboratory animals	Female nod-scid mice and female Balb/c OlaHsd mice were used from 8 to 12 weeks of age.
Wild animals	The study did not involve wild animals
Field-collected samples	The study did not involve field-collected sample
Ethics oversight	All animal experiments were performed in accordance with the guidelines established in the Principles of Laboratory Animal Care (directive 86/609/EEC), were approved by the Italian Ministry of Health and were performed under the supervision of the institutional organism for animal welfare (Cogentech OPBA). For the orthotopic model, the animal protocol was approved by the Institutional Animal Care and Use Committee (IACUC) of the University of Southern California.

Note that full information on the approval of the study protocol must also be provided in the manuscript.

Human research participants

Policy information about studies involving human research participants

Population characteristics	TCGA-COAD patient information, including gender, mutational status and clinical data, could be retrieved through GDC Data Portal (https://portal.gdc.cancer.gov/projects/TCGA-COAD).
Recruitment	Molecular data and clinical information including KRAS status and, survival and RNA-seq FTH1 mRNA expression data in TCGA-COAD (n =266) samples were collected from GDC Data Portal (https://portal.gdc.cancer.gov) and TSVdb (Sun et al., 2018) respectively. Patients with undefined survival time or without available RNA-seq data were excluded, only primary solid tumors were considered. KRAS-mutant tumors were selected according to the presence of a predicted deleterious mutation (DH; SIFT value <0.05).
Ethics oversight	Sequencing of human subjects' tissue was performed by TCGA consortium members under a series of locally approved Institutional Review Board (IRB) protocols as described in (Cancer Genome Atlas Network (2012)). Informed consent was obtained from all human participants.

Note that full information on the approval of the study protocol must also be provided in the manuscript.

Flow Cytometry

Plots

Confirm that:

- [x] The axis labels state the marker and fluorochrome used (e.g. CD4-FITC).
- [x] The axis scales are clearly visible. Include numbers along axes only for bottom left plot of group (a 'group' is an analysis of identical markers).
- [x] All plots are contour plots with outliers or pseudocolor plots.
- [x] A numerical value for number of cells or percentage (with statistics) is provided.

Methodology

Sample preparation	All cell lines were processed according the manufacturer's protocol. Cells were were trypsinized, resuspended in their respective media, and stained with CellROX deep red reagent (1 μM) in the dark (with or without test compound), for 30 minutes at 37°C and 5% CO2. Then, fluorescence was immediately analyzed by flow cytometry.
Instrument	Flow cytometer (Attune NxT flow cytometer)
Software	Data were processed by Kaluza analysis software (Beckman coulter, version 2.0)
Cell population abundance	The entire cell population was analyzed without post-sort analysis for each cell line
Gating strategy	Cells were first gated based on the size (FSC) and on density and granularity of the cells (SSC), excluding debris and doublets; only the live fraction was considered.<br>Then, a first gate was applied on the basis of not-stained negative control cells to identified stained-positive cells.<br>Next, a second gate was applied above the APC fluorescence level of the control condition (growing cells/ no-treated). The fluorescence intensity of treated samples was compared to the fluorescence intensity of control sample.<br>An example of gating strategy is provided.

- [x] Tick this box to confirm that a figure exemplifying the gating strategy is provided in the Supplementary Information.
